# Nuclear Magnetic Resonance-Based Metabolomics to Predict Early and Late Adverse Outcomes in Ischemic Stroke Treated with Intravenous Thrombolysis

**DOI:** 10.1021/acs.jproteome.2c00333

**Published:** 2022-12-05

**Authors:** Cristina Licari, Leonardo Tenori, Francesca Di Cesare, Claudio Luchinat, Betti Giusti, Ada Kura, Rosina De Cario, Domenico Inzitari, Benedetta Piccardi, Mascia Nesi, Cristina Sarti, Francesco Arba, Vanessa Palumbo, Patrizia Nencini, Rossella Marcucci, Anna Maria Gori, Elena Sticchi

**Affiliations:** † Magnetic Resonance Center (CERM), University of Florence, Via Luigi Sacconi 6, Sesto Fiorentino, Firenze 50019, Italy; ‡ Department of Chemistry “Ugo Schiff”, University of Florence, Via della Lastruccia 3-13, Sesto Fiorentino, Florence 50019, Italy; § CIRMMP, Via Luigi Sacconi 6, Sesto Fiorentino, Florence 50019, Italy; ∥ Department of Experimental and Clinical Medicine, University of Florence, Largo Brambilla 3, Florence 50134, Italy; ⊥ Atherothrombotic Diseases Center, Careggi Hospital, Florence, Largo Brambilla 3, Florence 50134, Italy; # Excellence Centre for Research, Transfer and High Education for the Development of DE NOVO Therapies (DENOTHE), University of Florence, Viale Pieraccini 6, Firenze 50139, Italy; ¶ Stroke Unit, Careggi University Hospital, Florence 50134, Italy; ∇ Institute of Neuroscience, Italian National Research Council (CNR), Via Madonna del Piano, 10, Sesto Fiorentino, Florence 50019, Italy; ○ NEUROFARBA Department, Neuroscience Section, University of Florence, Largo Brambilla 3, Florence 50134, Italy; ⧫ Department of Neurology, Careggi University Hospital, Largo Brambilla 3, Florence 50134, Italy

**Keywords:** ischemic stroke, metabolomics, lipoproteomics, nuclear magnetic resonance

## Abstract

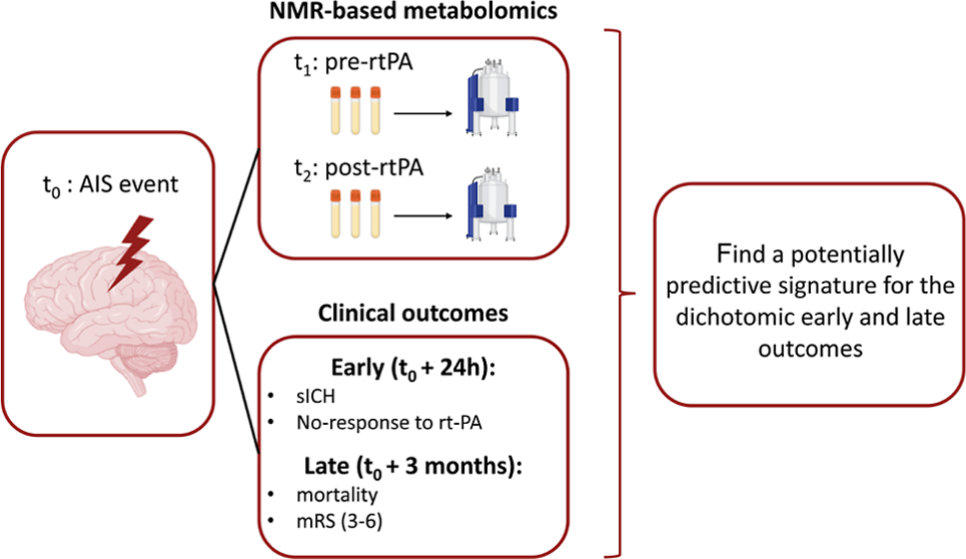

Metabolic perturbations and inflammatory mediators play a fundamental role in both early and late adverse post-acute ischemic stroke outcomes. Using data from the observational MAGIC (MArker bioloGici nell’Ictus Cerebrale) study, we evaluated the effect of 130 serum metabolic features, using a nuclear magnetic spectroscopy approach, on the following outcomes: hemorrhagic transformation at 24 h after stroke, non-response to intravenous thrombolytic treatment with the recombinant tissue plasminogen activator (rt-PA), and the 3 month functional outcome. Blood circulating metabolites, lipoproteins, and inflammatory markers were assessed at the baseline and 24 h after rt-PA treatment. Adjusting for the major determinants for unfavorable outcomes (i.e., age, sex, time onset-to-treatment, etc.), we found that acetone and 3-hydroxybutyrate were associated with symptomatic hemorrhagic transformation and with non-response to rt-PA; while 24 h after rt-PA, levels of triglycerides high-density lipoprotein (HDL) and triglycerides low-density lipoprotein (LDL) were associated with 3 month mortality. Cholesterol and phospholipids levels, mainly related to smaller and denser very low-density lipoprotein (VLDL) and LDL subfractions were associated with 3 month poor functional outcomes. We also reported associations between baseline 24 h relative variation (Δ) in VLDL subfractions and ΔC-reactive protein, Δinterleukin-10 levels with hemorrhagic transformation. All observed metabolic changes reflect a general condition of energy failure, oxidative stress, and systemic inflammation that characterize the development of adverse outcomes.

## 1 Introduction

Ischemic stroke is a leading cause of death and disability continuously increasing^1^ and contributing significantly to health costs. There is an urgent need to find biomarkers useful for clinical practice and to better understand metabolic dysregulation on the basis of the pathophysiological mechanisms of the disease. In this light, metabolic perturbations are fundamental events that contribute to ischemic stroke, its progression, and the development of unfavorable outcomes.^2−5^ Regarding the knowledge about biomarkers associated with poor prognosis in the setting of stroke patients treated with thrombolysis, little evidence has been reported (i.e., glucose).^6^ Dyslipidaemia is a risk factor contributing to the onset of ischemic stroke; high levels of total cholesterol and low-density lipoprotein (LDL) cholesterol increase the risk for cerebral ischemia.^7,8^ However, the effects of lipid levels on clinical outcomes after the ischemic attack are controversial: high total cholesterol and LDL levels have been associated with better functional and vital outcomes after stroke,^9,10^ while low LDL levels increased the risk of early symptomatic intracranial hemorrhage^11^ and low total cholesterol was related to a worse functional outcome in ischemic stroke patients after the thrombolytic treatment.^12^ Therefore, the effective contribution of lipid levels to stroke outcomes, particularly after thrombolysis, needs to be further investigated.

In this framework, nuclear magnetic resonance (NMR)-based metabolomics can provide crucial information, allowing a high-throughput analysis of various types of samples (i.e., blood and urine) and giving information on various molecular features present in biological matrices.^13,14^ Here, using data of patients enrolled in the MAGIC (MArker bioloGici nell’Ictus Cerebrale) study,^15−17^ we aimed at providing metabolic insights underlying susceptibility to early and late post-acute ischemic stroke (AIS) adverse outcomes.

We also studied the interplay between statistically significant metabolomic features and circulating inflammatory markers, such as Δ of metalloproteinase 9 (MMP9), alpha2 macroglobulin (A2M), serum amyloid protein (SAP), C reactive protein (CRP), interleukins (ILs), tumor necrosis factor alpha (TNFα), and monocyte chemo-attractant protein I (MCPI), resulted to be statistically associated to secondary intracerebral hemorrhage (sICH), 3 month mortality, and 3 month poor functional outcome, as previously described in the publication of Gori et al.^16^

**Figure 1:**
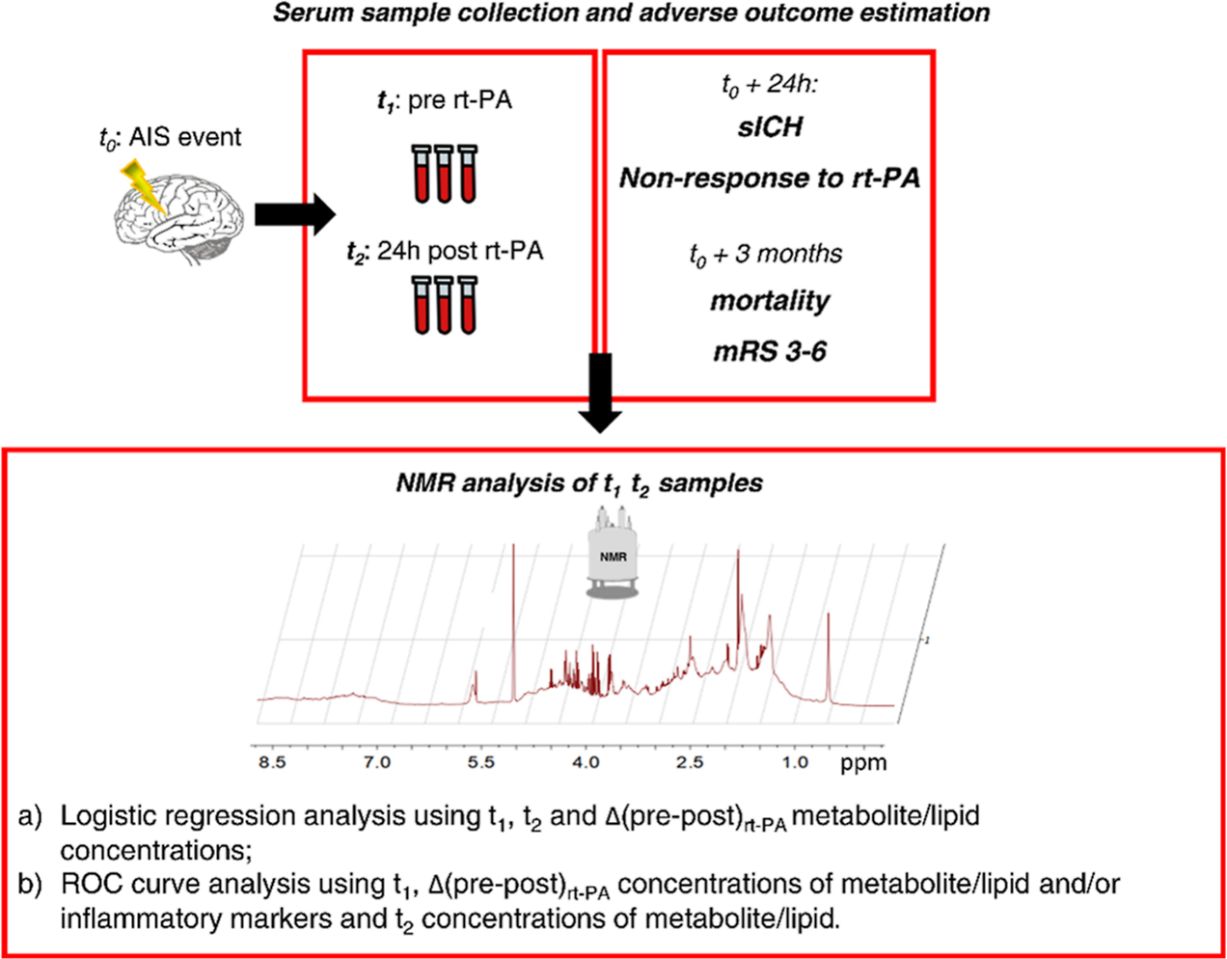
Graphical representation of the analysis followed to identify, in serum samples, possible predictors of early and late adverse outcomes of AIS treated with intravenous thrombolysis with rt-PA. Blood samples were collected before (*t*_1_) and 24 h after (*t*_2_) the administration of rt-PA. Early adverse outcomes were defined 24 h after the event, as follows: development of sICH and non-response to the intravenous thrombolysis. Late outcomes [mortality and disability (mRS 3–6)] were defined 3 months after the transient ischemia. For each time point, 1D NMR spectra have been acquired and therefore used to estimate metabolite and lipid concentrations; *t*_1_, *t*_2,_ and Δ(pre–post)_rt-PA_ concentrations of metabolites/lipoprotein.

An overview of the present study design is reported in Figure 1.

## 2 Materials and Methods

### 2.1 Study Population and Outcomes

The subjects and the study samples considered are from the MAGIC study^15,16^ in which 327 patients were enrolled; only subjects for whom serum aliquots were available for metabolomics analysis are considered in this study (*n* = 243).

The study population considered here is characterized by patients who had an AIS and were admitted for thrombolysis treatment with the recombinant tissue plasminogen activator (rt-PA), in 14 different Italian centers, registered in the Safe Implementation of Thrombolysis in Stroke International Stroke Thrombolysis Register (SITS-ISTR, www.sitsinternational.org), according to the SITS-Monitoring Study criteria,^18^ in the frame of the national, observational, and multicentric MAGIC study.^15,16^

The whole study focuses on the analysis of serum samples collected at two different time points: before (*t*_1_) and 24 h after (*t*_2_) the administration of rt-PA. Adverse outcomes were defined as follows: (i) early outcomes (24 h) characterized by development of symptomatic intracerebral hemorrhage (sICH), according to the National Institute of Neurological Disorders and Stroke criteria,^19^ and the absence of clinical response to systemic thrombolysis [<4 point decrease on 24 h National Institutes of Health Stroke Scale (NIHSS)] and (ii) late outcomes (3 months) characterized by death and disability defined as the modified Rankin scale (mRS), dichotomized into a good (mRS, 0–2) or poor (mRS, 3–6) functional status.

A description of the clinical and demographic characteristics of the patients is reported in Table 1.

**Table 1:** Demographic and Clinical Characteristics of the 243 Patients Selected for This Study^a^

demographics	*n* = 243
age, years, mean and SD	68.8 ± 11.9
sex (male), *n* (%)	137 (56.4%)
onset to treatment time, minutes, mean and SD	163.4 ± 83.7
NIHSS, mean and SD	11.9 ± 6.1
baseline systolic blood pressure, mmHg, mean and SD	147.5 ± 21.3
baseline diastolic blood pressure, mmHg, mean and SD	79.7 ± 12.7
blood glucose, mg/dL, mean and SD	130.2 ± 49.5
Risk Factors	
hypertension, *n* (%)	143 (58.8%)
diabetes, *n* (%)	36 (14.8%)
hyperlipidaemia, *n* (%)	56 (23%)
current smoking, *n* (%)	35 (14.4%)
atrial fibrillation, *n* (%)	56 (23%)
congestive heart failure, *n* (%)	26 (10.7%)

a Main abbreviations: SD: standard deviation and NIHSS: National Institutes of Health Stroke Scale.

### 2.2 Ethical Issues

The study protocol was approved by the local Ethical Committee of each participating Centre, which complies with the Declaration of Helsinki. All patients gave informed consent.

### 2.3 NMR Sample Collection and Preparation

Whole venous blood was collected in tubes without an anticoagulant, before and 24 h after thrombolysis. Tubes were centrifuged at room temperature at 1500*g* for 15 min, and the supernatants were stored in aliquots at −80 °C until NMR measurements.

For metabolomic analyses, serum samples were prepared following the details reported elsewhere.^13^

### 2.4 NMR Experiments

Serum samples were analyzed using a Bruker 600 MHz spectrometer working at a 600.13 MHz proton Larmor frequency equipped with a 5 mm PATXI ^1^H–^13^C–^15^N and ^2^H decoupling probe. This includes a *z*-axis gradient coil, an automatic tuning–matching, and an automatic and refrigerated sample changer (SampleJet). To stabilize approximately at the level of ±0.1 K, the sample temperature (310 K), a BTO 2000 thermocouple was employed and each NMR tube was kept for at least 5 min inside the NMR probe head to equilibrate the acquisition temperature of 310 K.

For each serum specimen, three one-dimensional proton NMR spectra [i.e., 1D nuclear Overhauser effect spectroscopy (NOESY), 1D Carr–Purcell–Meiboom–Gill, and 1D diffusion-edited] were acquired with different pulse sequences,^20−22^ allowing the selective detection of different molecular components. Detailed procedures on parameters of NMR experiments are reported elsewhere.^13^

Raw NMR data were multiplied by an exponential function of 0.3 Hz line-broadening factor before the application of Fourier transform. Phase and baseline distortions were automatically corrected, and transformed spectra were calibrated to the glucose doublet at 5.24 ppm using TopSpin 3.2 (BrukerBioSpin).

### 2.5 Metabolite and Lipoprotein Identification and Quantification

18 metabolites and 112 lipoproteins were identified and estimated from ^1^H 1D NOESY NMR spectra according to Bruker’s B.I.-LISA protocols.^23^ A complete list of the molecular features analyzed in this study is presented in Table S1.

### 2.6 Laboratory Measurements

Levels of different inflammatory markers(IL, TNFα, CRP, A2M, SAP, and MCPI) have been measured, as previously reported in Gori et al.^16^

### 2.7 Statistical Analysis

#### 2.7.1 Demographic and Clinics

For demographics, clinical characteristics, and risk factors, a *t*-test and a χ-square test were applied, respectively, for comparisons including continuous variables and categorical variables.

#### 2.7.2 Exploratory Analysis

Principal component analysis (PCA)^24,25^ was performed to explore data patterns. Data were scaled to unit variance before analysis.

#### 2.7.3 Correlation Analysis

Pearson correlation analysis^26,27^ was used to study the potential association between inflammatory markers and metabolomics data at *t*_1_ and *t*_2_. The significance threshold has been imposed at a *P*-value of < 0.05. For completeness, adjusted *P*-values, using the Benjamini–Hochberg method,^28^ were also reported.

#### 2.7.4 Penalized Logistic Regression Analyses

As main explanatory variables, we considered the baseline (*t*_1_), 24 h post (*t*_2_) rt-PA, and a single patient’s relative pre-24 h and post-rt-PA variation [Δ(pre–post)_rt-PA_] of metabolites and lipid concentrations.

For each metabolite or lipid *x*, the variation in terms of concentration between the time points *t*_1_ and *t*_2_ was calculated as follows
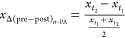
1

The statistically robust effect of each variable at *t*_1_, *t*_2_, or considering Δ(pre–post)_rt-PA_ on the four adverse outcomes was estimated by penalized logistic regression analysis,^29^ including as covariates different patients’ characteristics, that is, age, sex, baseline, 24 h post-blood glucose (for *t*_1_ and *t*_2_ models, respectively), baseline NIHSS, time onset-to treatment, blood collection center, risk factors, and comorbidities (i.e., history of atrial fibrillation, congestive heart failure, recent infections or inflammations, hypertension, diabetes, hyperlipidaemia, and smoke).

Odd ratio (OR) values and 95% confidence interval (95% CI) were reported for each metabolite or lipoprotein analyzed. For completeness, adjusted *P*-values, obtained using the Benjamini–Hochberg method,^28^ were also reported. Since correction for multiple testing increases the risk of false a negative, especially in the case where (possibly) weak associations are tested on a large number of variables, we presented both corrected and uncorrected *P*-values. We considered significant *P*-values < 0.05.

#### 2.7.5 Receiver Operating Characteristic Curve Analysis

Adverse outcomes were evaluated also by applying receiver operating characteristic (ROC) curve analysis on selected analytes at *t*_1_ and *t*_2_ and considering specific patient’s relative metabolites and lipids Δ(pre–post)_rt-PA_ concentrations.

In detail, for each of the evaluated outcomes, we estimated the values of the area under the ROC curve (AUC-ROC) for two different penalized logistic regression models. First, we calculated AUC values for models [hereafter referred as “Bas1” for *t*_1_ and Δ(pre–post)_rt-PA,_ and “Bas2” for *t*_2_] that included only clinical and risk factors known to affect the outcomes (i.e., age, gender, blood collection center, time onset-to-treatment, recent infections or inflammations, glycemia, NIHSS, history of atrial fibrillation, and congestive heart failure). Second, we quantify how much the addition of a combination of metabolomic features increases the prediction for events when added to each “Bas” ROC curve model. The metabolites and/or lipoproteins to be included are chosen among the top three statistically significant features based on the results of the penalized logistic regression models.

Third, for *t*_1_ and Δ(pre–post)_rt-PA_ ROC models, we combined statistically significant metabolic features with specific blood circulating inflammatory markers statistically associated with sICH, 3 month mortality, and 3 month poor functional outcome (mRS = 3–6), selecting them based on the results reported in the original publication of Gori et al.^16^

For all ROC models, 95% CIs of the AUC values have been calculated, together with a *P*-value, to highlight any significant changes in the prediction of the adverse outcome after considering the association of metabolomics and clinical features.

To avoid overfitting, before performing any ROC analysis, all penalized logistic regression models were cross-validated using the leave-one-out scheme.

#### 2.7.6 Software

All statistical analyses were performed using R (version 3.5.3), an open-source software for the statistical management of data.^30^ Penalized logistic regression models were built using the logistf function of the R package “logistf”.^29,31^ To perform ROC analysis roc function of the R package, “pROC” was used.^32^ To perform correlation analysis, the rcorr function of the R package “Hmisc” was used. The plot was generated using the R package “ggplot2”.^33^

## 3 Results

### 3.1 Exploration Analysis and Association between Molecular Features and Inflammatory Markers

**Figure 2:**
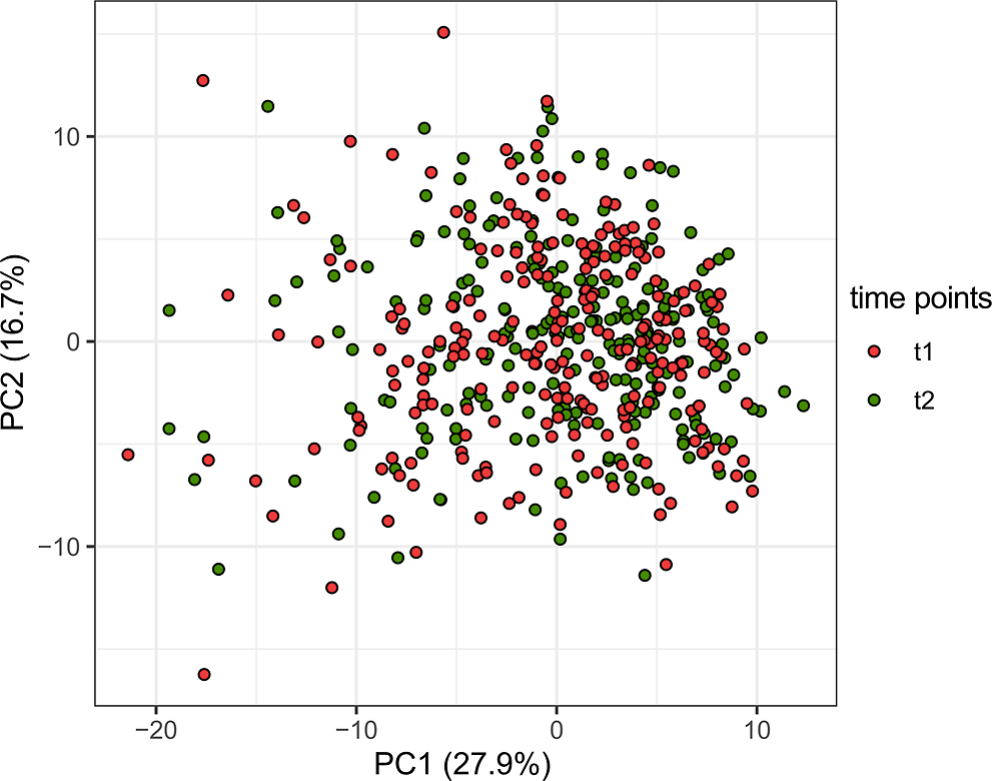
Scatter plot of PCA, taking into account the first two principal components. The red dots represent the serum metabolic profiles collected before (*t*_1_) the administration of rt-PA, and the green dots represent the serum metabolic profiles collected 24 h after (*t*_2_) the administration of rt-PA.

All analyses have been performed using data from 243 patients of the original cohort belonging to the MAGIC study.^15,16^ Notwithstanding this, all demographics, clinical characteristics, and risk factors do not statistically change (*P*-value > 0.05) between the original cohort and the sub-cohorts analyzed in this study (see Table S2). First, as a multivariate exploratory approach, PCA was performed using all 18 metabolites and 112 lipoproteins detected on *n* = 243 serum NMR spectra in order to obtain an overview of the variation in the data and to check the presence of metabolic signatures among the different evaluated conditions. As shown in Figure 2, there is no separation among the samples collected before (*t*_1_) and 24 h after (*t*_2_) the administration of rt-PA, and no outliers are highlighted, confirming the good quality of the data.

To highlight potential associations between molecular features and inflammatory markers before (*t*_1_) and 24 h after (*t*_2_) rt-PA, Pearson correlation analysis was also performed. In Tables S3 and S4, only the significant correlations (*P*-value < 0.05) were reported, respectively, for *t*_1_ and *t*_2_ time points. Before the administration of rt-PA (*t*_1_) we observed, in particular, correlations between lipoproteins and IL-8 and CRP; after the administration of rt-PA (*t*_2_), we observed, in particular, correlations between lipoproteins and IL-6, IL-12 and CRP. It is important to underline that no strong correlations (Pearson’s coefficient *r*> |0.6| were observed.

### 3.2 Molecular Features Associated with Early Adverse Outcomes

#### 3.2.1 Development of sICH

Before starting the thrombolytic therapy (*t*_1_), acetone and 3-hydroxybutyrate were determined to be the only metabolites significantly (*P*-values < 0.05) associated with the development of symptomatic intracranial hemorrhage. At *t*_2_, 18 lipids out of 112 were associated with the evaluated outcome, especially lipids related to bigger and less dense very low-density lipoprotein (VLDL) particles. Considering Δ(pre–post)_rt-PA_ metabolite/lipid concentrations, we reported phenylalanine, pyruvate, and glucose to be the most statistically associated metabolites, while among lipid parameters, phospholipids related to VLDL particles are the most statistically associated analytes (see Table 2). To view the complete lists of all metabolites and lipids effects at *t*_1_, *t*_2_, and Δ(pre–post)_rt-PA_, we refer the reader to the Supporting Information (Tables S5–S7).

**Table 2:** Effect of Statistically Significantly (*P*-Values < 0.05) Associated Pre- (*t*_1_), 24 h Post- (*t*_2_), and Δ(Pre–Post) rt-PA Molecular Features Concentrations on Early (sICH, Non-response to Thrombolysis) and Late (3 Month Mortality and 3 Month Poor Functional Outcome) Adverse Outcomes, Adjusting for Major Determinants for Unfavorable Outcomes, That Is, Age, Sex, Time Onset-to-Treatment, Pre- or 24 h Post-rt-PA Blood Glucose Level (for *t*_1_ and *t*_2_ Models, Respectively), Baseline NIHSS, History of Atrial Fibrillation, Congestive Heart Failure, Recent Infections or Inflammations, Hypertension, Diabetes, Hyperlipidaemia, Smoke, and Blood Collection Center^a^

	pre rt-PA (*t*_1_)				24 h post rt-PA (*t*_2_)				Δ(pre–post)_rt-PA_			
	analytes	OR (95% CI)	P	FDR	analytes	OR (95% CI)	P	FDR	analytes	OR (95% CI)	P	FDR
												

(sICH)												
early outcomes	3-HB	1.457 (1.062–1.998)	0.025	0.455	SubPhosp_VLDL-2	0.364 (0.193–0.687)	0.001	0.097	SubPhosp_VLDL-2	0.501 (0.323–0.777)	0.004	0.301
	acetone	1.393 (1.016–1.909)	0.046	0.502	SubTrigl_VLDL-2	0.372 (0.193–0.717)	0.003	0.097	LMF_Phosp_VLDL	0.525 (0.339–0.814)	0.006	0.301
					SubChol_VLDL-2	0.383 (0.203–0.719)	0.003	0.097	SubChol_VLDL-2	0.554 (0.366–0.838)	0.009	0.301
					LMF_Phosp_VLDL	0.382 (0.194–0.753)	0.006	0.128	SubPhosp_VLDL-3	0.534 (0.332–0.86)	0.014	0.301
					SubTrigl_VLDL-3	0.44 (0.242–0.803)	0.008	0.128	Phe	1.736 (1.157–2.605)	0.015	0.274
					LMF_FreeChol_VLDL	0.419 (0.222–0.793)	0.009	0.128	SubTrigl_HDL-3	0.626 (0.43–0.912)	0.019	0.301
					LMF_Chol_VLDL	0.452 (0.251–0.812)	0.010	0.128	SubFreeChol_VLDL-1	0.57 (0.363–0.896)	0.020	0.301
					SubPhosp_VLDL-3	0.46 (0.257–0.822)	0.010	0.128	LMF_FreeChol_VLDL	0.602 (0.399–0.91)	0.023	0.301
					SubChol_VLDL-3	0.465 (0.262–0.825)	0.010	0.128	VLDL_PN	0.597 (0.39–0.914)	0.024	0.301
					SubFreeChol_VLDL-2	0.46 (0.249–0.852)	0.014	0.164	LMF_ApoB_VLDL	0.597 (0.39–0.914)	0.024	0.301
					SubPhosp_VLDL-1	0.361 (0.157–0.832)	0.017	0.171	LMF_Chol_VLDL	0.611 (0.405–0.921)	0.026	0.301
					SubFreeChol_VLDL-3	0.468 (0.253–0.868)	0.018	0.171	pyruvic acid	1.692 (1.062–2.695)	0.035	0.297
					LMF_Trigl_VLDL	0.405 (0.19–0.867)	0.020	0.178	SubTrigl_HDL-4	0.656 (0.449–0.959)	0.037	0.339
					SubChol_VLDL-1	0.395 (0.179–0.869)	0.024	0.194	SubPhosp_VLDL-4	0.627 (0.412–0.952)	0.039	0.339
					SubFreeChol_VLDL-1	0.424 (0.195–0.923)	0.031	0.237	SubTrigl_VLDL-2	0.608 (0.392–0.945)	0.041	0.339
					SubChol_VLDL-4	0.575 (0.349–0.947)	0.038	0.270	LMF_Trigl_IDL	0.609 (0.383–0.97)	0.047	0.339
					LMF_Trigl_IDL	0.487 (0.247–0.962)	0.040	0.271	SubTrigl_VLDL-4	0.602 (0.372–0.973)	0.047	0.339
					Trigl	0.483 (0.241–0.968)	0.044	0.276	glucose	1.68 (1.042–2.707)	0.050	0.297

(Non-Response to Thrombolysis)												
	acetone	0.696 (0.509–0.95)	0.017	0.219	3-HB	0.571 (0.413–0.791)	0.0004	0.007	acetone	0.684 (0.516–0.906)	0.007	0.129
	3-HB	0.718 (0.531–0.97)	0.024	0.219	acetone	0.609 (0.43–0.863)	0.003	0.026	SubTrigl_HDL-4	1.386 (1.026–1.872)	0.028	0.238
	LMF_Trigl_HDL	1.375 (1.016–1.862)	0.036	0.956(Three-Month Mortality)	SubTrigl_HDL-4	1.559 (1.135–2.143)	0.005	0.586	3-HB	0.725 (0.543–0.969)	0.028	0.238
	SubTrigl_HDL-3	1.343 (0.998–1.806)	0.049	0.956	Phe	0.722 (0.542–0.962)	0.024	0.143	creatinine	0.755 (0.568–1.003)	0.047	0.238

(Three-Month Mortality)												
late outcomes	SubTrigl_HDL-3	0.61 (0.372–1.002	0.045	0.994	SubTrigl_LDL-6	1.751 (1.182–2.594)	0.010	0.982	LMF_Trigl_LDL	0.609 (0.414–0.897)	0.023	0.934
					SubChol_LDL-6	1.804 (1.084–3.003)	0.042	0.982	LMF_Trigl_HDL	0.597 (0.404–0.882)	0.031	0.934
					LMF_Trigl_HDL	0.526 (0.299–0.927)	0.048	0.982	SubTrigl_LDL-2	0.567 (0.353–0.912)	0.039	0.934
									SubTrigl_HDL-4	0.634 (0.429–0.937)	0.040	0.934
									SubTrigl_LDL-1	0.576 (0.357–0.927)	0.041	0.934

(Three-Month Poor Functional Outcome)												
	SubPhosp_HDL-3	0.549 (0.365–0.826)	0.004	0.209	SubChol_VLDL-5	0.497 (0.334–0.738)	0.0005	0.040	Glu	1.565 (1.069–2.29)	0.023	0.412
	Apo-B100-Apo-A1	1.63 (1.131–2.349)	0.007	0.209	SubTrigl_LDL-6	1.745 (1.213–2.508)	0.001	0.040	SubApoA2_HDL-2	1.419 (1.004–2.006)	0.042	0.973
	LMF_Phosp_HDL	0.584 (0.393–0.867)	0.008	0.209	SubFreeChol_LDL-6	1.844 (1.266–2.687)	0.001	0.040	SubApoA2_HDL-1	1.38 (0.978–1.946)	0.044	0.973
	SubChol_VLDL-5	0.618 (0.427–0.893)	0.011	0.209	LDL6_PN	1.79 (1.233–2.597)	0.002	0.040				
	SubFreeChol_LDL-6	1.575 (1.116–2.221)	0.011	0.209	SubApoB_LDL-6	1.79 (1.233–2.597)	0.002	0.040				
	SubPhosp_VLDL-5	0.641 (0.444–0.926)	0.019	0.268	SubChol_LDL-6	1.743 (1.207–2.516)	0.003	0.049				
	SubPhosp_HDL-2	0.633 (0.429–0.934)	0.022	0.268	SubPhosp_LDL-6	1.701 (1.184–2.445)	0.004	0.061				
	SubTrigl_LDL-6	1.464 (1.059–2.025)	0.024	0.268	SubPhosp_VLDL-5	0.585 (0.401–0.852)	0.006	0.076				
	SubApoA1_HDL-3	0.637 (0.43–0.944)	0.025	0.268	Apo-B100-Apo-A1	1.738 (1.17–2.583)	0.006	0.076				
	SubApoA1_HDL-2	0.644 (0.438–0.946)	0.026	0.268	Acetic acid	1.651 (1.133–2.407)	0.010	0.173				
	SubChol_LDL-6	1.461 (1.049–2.035)	0.029	0.280	Apo-B100	1.595 (1.087–2.339)	0.018	0.175				
	LMF_ApoA1_HDL	0.674 (0.465–0.978)	0.040	0.331	LMF_ApoB_LDL	1.524 (1.044–2.226)	0.031	0.246				
	SubApoB_LDL-6	1.418 (1.022–1.967)	0.044	0.331	LDL_PN	1.524 (1.044–2.226)	0.031	0.246				
	LDL6_PN	1.418 (1.022–1.967)	0.044	0.331	LMF_FreeChol_LDL	1.532 (1.043–2.251)	0.033	0.246				
					LMF_Chol_IDL	1.5 (1.034–2.177)	0.037	0.246				
					SubTrigl_VLDL-1	1.533 (1.044–2.251)	0.037	0.246				
					LMF_Phosp_HDL	0.669 (0.458–0.977)	0.041	0.257				
					3-HB	1.434 (1.013–2.032)	0.045	0.403				

a Main abbreviations: NIHSS: National Institutes of Health Stroke Scale; OR: odds ratio; CI: confidence interval; P: *P*-values; trigl: triglycerides; chol: cholesterol; phosp: phospholipids; Apo: apolipoprotein; LMF: lipoprotein main fraction; Sub: subfraction; PN: particle number; and 3-HB: 3-hydroxybutyrate. Amino acids are reported with the three letter code.

Considering these results, as reported in Table 3, for the *t*_1_ time point, the addition of acetone and 3-hydroxybutyrate to the Bas1 ROC curve model did not statistically improve the AUC value (model Bas1: AUC = 0.58 and model Bas1 + G: AUC = 0.59, *P*-value = 0.349). Adding baseline levels of the interleukin I receptor antagonist (IL-IRa) and IL-10 to Bas1 + G model, the resulting model got worse (model Bas1 + G + P: AUC = 0.51, *P*-value = 0.87).

**Table 3:**
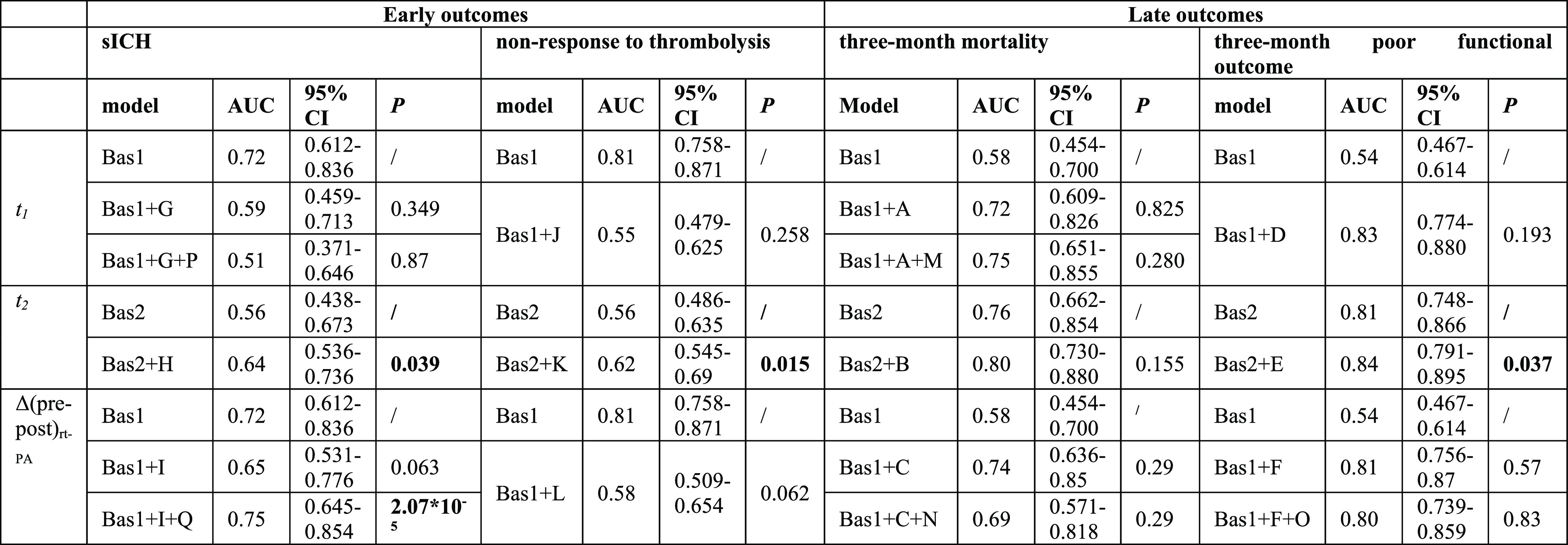
ROC Curve Models For *t*_1_, *t*_2_, and Δ(pre-post)_rt-PA_, Metabolites/Lipids Values Considering Early (sICH, Non-Response to Thrombolysis) and Late (Three-Month Mortality and Three-Month Poor Functional Outcome) Outcomes. AUC Values Are Reported Both for Models Built Considering Only Clinical Characteristics, Risk Factors, And Comorbidities (Referred to As Bas1 or Bas2 for t1, Δ(pre-post)_rt-PA_, and *t*_2_ Samples, Respectively), And for Models Obtained after Adding Selected Combinations among the Top Three Statistically Significant Metabolomic Features, Selected from Previous Penalized Logistic Regression Analysis; Models Obtained after Combining Metabolic Features and Circulating Inflammatory Markers Statistically Associated with sICH, Mortality, And Poor Functional Outcome Are Also Described. 95% CIs And *P*-values (*P*) Are Also Reported^a^

a Abbreviations used: Bas1: correction for age, gender, blood collection center, time onset-to-treatment, recent infections or inflammations, baseline values of glycemia, NIHSS, history of atrial fibrillation, and congestive heart failure; Bas2: correction for age, gender, blood collection center, time onset-to-treatment, 24h post rt-PA glycemia, recent infections or inflammations, baseline values of NIHSS, history of atrial fibrillation, and congestive heart failure. A: DL-3 triglycerides; B: LDL-6 triglycerides, HDL triglycerides, LDL-6 cholesterol; C: main fraction triglycerides LDL, HDL, LDL-2 triglycerides; D: HDL-3 phospholipids, HDL phospholipids main fraction, HDL/LDL cholesterol; E: VLDL-5 cholesterol, LDL-6 free cholesterol, LDL-6 triglycerides; F: glutamate, HDL-1 apolipoprotein A2, HDL-2 apolipoprotein A2; G: 3-HB, acetone; H: VLDL-2 cholesterol/phospholipids/triglycerides; I: main fraction VLDL phospholipids, VLDL-2 cholesterol, VLDL-2 phospholipids; J: 3-HB, acetone; K: 3-hydroxybutyrate, acetone; L: acetone, LDL-3 particle number, LDL-3 apolipoprotein B; M: ΔMMP9, SAP, and A2M; N: ΔIL-IRa, ΔIL-10, and ΔTNFα; O: ΔIL-6, ΔIL-8, ΔIL-10, ΔIL-12, ΔMCPI, ΔTNFα, and ΔCRP; and P: IL-Ira, IL-10; Q: ΔCRP, and ΔIL-10.

For the *t*_2_ time point, the addition of VLDL-2 cholesterol, phospholipids, and triglycerides to the Bas2 model statistically increased the baseline AUC value of 0.556 to a value of 0.636 (*P*-value = 0.039, model Bas2 + H).

Finally, considering Δ(pre–post)_rt-PA_ metabolic feature concentrations, adding the values related to VLDL phospholipid main fraction and the ones related to the VLDL-2 cholesterol and phospholipid subfractions to the Bas 1 ROC model, we obtained an improvement of the related AUC value, changing from 0.58 to a value of 0.65 (*P*-value = 0.063, model Bas1 + I). Moreover, ROC analysis demonstrated that the addition of ΔCRP and ΔIL-10 to the Bas1 + I model significantly improved the AUC for the prediction of sICH in AIS patients treated with thrombolysis (model Bas1 + I + Q: AUC = 0.75, *P*-value = 2.07 × 10^–5^).

#### 3.2.2 Non-response to the Intravenous Thrombolytic Therapy

At the *t*_1_ time point, acetone and 3-hydroxybutyrate were determined to be associated with the non-response to the intravenous thrombolysis. At *t*_2_, we estimated the association for phenylalanine, VLDL-5 phospholipids, high-density lipoprotein (HDL)-4 triglycerides, HDL-2 free cholesterol, acetone, and 3-hydroxybutyrate. About Δ(pre–post)_rt-PA_, alanine, acetone, the total particle number of Apo-B100, related LDL-3 sub-particles, and HDL-4 triglycerides were the significant predictors of non-response to the therapeutic thrombolytic treatment (see Tables 2, and S5–S7).

As reported in Table 3, at *t*_1_, the addiction of acetone and 3-hydroxybutyrate to the baseline Bas1 ROC curve model did not statistically improve the AUC (model Bas1: AUC = 0.54 and model Bas1 + J: AUC = 0.55). At *t*_2_, adding the same two ketone bodies, also reporting FDR values < 0.05, to the Bas2 ROC curve model, the AUC significantly increased to a value of 0.62 (model Bas2 + K). Regarding Δ(pre–post)_rt-PA_, the addition of acetone, the LDL-3 particle number, and LDL-3 apolipoprotein-B to the respective Bas1 ROC curve model led to a slight and not significant improvement of the AUC, changing from 0.54 to 0.58 (model Bas1 + L).

### 3.3 Molecular Features Associated with Late Adverse Outcomes

#### 3.3.1 3 Month Mortality

As reported in Table 2, triglycerides related to the HDL-3 subfraction are the only analytes presenting an association with the AIS patients’ 3 month mortality at *t*_1_. For *t*_2_, HDL triglycerides, LDL-6 related cholesterol, triglycerides and phospholipids, HDL-3, and HDL-4 triglycerides were determined associated with this late outcome. Considering Δ(pre-–post)_rt-PA_, triglycerides related to LDL and HDL particles and LDL-1, LDL-2, and HDL-4 sub-particles were determined associated with 3 month mortality (for more information see Tables S5–S7).

At *t*_1_, after adding triglycerides related to the HDL-3 subfraction to the Bas1 ROC model, the AUC did not improve (model Bas1 + A). Adding to this model levels of ΔMMP9, SAP, and A2M, a non-statistically significant improvement of the AUC was obtained (model Bas + A + M: AUC = 0.75, *P*-value = 0.28). At *t*_2_, after adding LDL-6, HDL-triglycerides, and LDL-6 cholesterol to the related baseline model, the AUC increased, without a statistical significance, from 0.76 to 0.80 (Bas2 + B). Lastly, considering Δ(pre–post)_rt-PA_, the addition of HDL, LDL, and LDL-2 triglycerides to the Bas1 ROC curve model increased, without significance, the AUC value from 0.72 to 0.74 (Bas1 + C). The Δ(pre–post) levels of IL-IRa, IL-10, and TNFα were added to the model Bas1 + C; the Bas1 + C + N model was determined to be worse (AUC = 69.5 and *P*-value = 0.29) (see Table 3).

#### 3.3.2 3 Month mRS 3–6

As reported in Table 2, at *t*_1_, 15 different lipids, mainly related to small dense VLDL, LDL, and HDL particles, were statistically associated with a 3 month poor functional outcome (mRS = 3–6). Considering *t*_2_ samples, acetic acid, 3-hydroxybutyrate, and 16 different lipids were determined to be statistically related to the development of poor functional outcomes (mRS = 3–6). Among the 16 lipids, triglycerides, total cholesterol, and free cholesterol estimated for LDL-6 subfractions appeared to be the most statistically significant associated metabolic features. When relating Δ(pre–post)_rt-PA_ metabolite and lipid concentrations to the 3 month poor functional outcome (mRS = 3–6), we found that glutamate, acetate, free cholesterol, phospholipids, and Apo A2, related mainly to HDL-1 and HDL-2, were statistically associated with the outcome (for more information, see Tables S5–S7).

As reported in Table 3, at *t*_1_, the addition of the top three statistically associated lipid fractions to the related baseline ROC model built at *t*_1_ did not statistically improve the AUC (model Bas1: AUC = 0.81 and model Bas1 + D AUC = 0.83, *P*-value = 0.193). For the ROC curve analysis, at *t*_2_, the addition of 24 h post rt-PA values of VLDL-5 cholesterol, LDL-6 free cholesterol, and LDL-6 triglycerides, reporting also FDR values < 0.05 in the penalized logistic regression, to the Bas2 ROC model, led to a statically significant improvement in the already good value of the baseline AUC (model Bas2: AUC = 0.807 and model Bas2 + E: AUC = 0.844, *P*-value = 0.037). Considering Δ(pre–post)_rt-PA_, adding glutamate and ApoA2, related to both HDL-1 and HDL-2, to the Bas1 ROC curve model, the AUC remained the same (model Bas1: AUC = 0.81 and model Bas1 + F: AUC = 0.81, *P*-value = 0.57). Lastly, adding to the Bas1 + F model Δ values of IL-6, IL-8, IL-10, IL-12, MCPI, TNFα, and CRP, the resulting Bas1 + F + O model, reported a similar value of the AUC (model Bas1 + F + O: AUC = 0.80, *P*-value = 0.83).

## 4 Discussion

We observed that various metabolomic features (especially lipids related to HDL, LDL, VLDL particles, and ketone bodies) resulted to be statistically associated (*P*-value < 0.05) with each of the assessed post-AIS adverse outcomes. Since correction for multiple testing increases the risk of false negatives, especially in the case where (possibly) weak associations are tested on a large number of variables, in this work, we present both corrected and uncorrected *P*-values and discussing the biological implications of the results for which *P*-values were significant before correction, improving the value of baseline AUC models for each specific outcome. Combining metabolomic features with inflammatory markers resulted in statistically significant associations among Δ levels of VLDL-2 cholesterol, VLDL-2 phospholipids, the main fraction of VLDL phospholipids, and ΔCRP, ΔIL-10 for the prediction of sICH.

We observed that acetone and 3-hydroxybutyrate appear to be involved in the symptomatic development of intracranial hemorrhage and in the non-response to the thrombolytic therapy, while *t*_2_ triglycerides levels, mainly associated with HDL and LDL particles, seem to be related to 3 month death. Moreover, alteration of cholesterol and phospholipids levels, mainly related to smaller and denser VLDL and LDL sub-particles, may be involved in the development of post-stroke impairments and neurological disabilities.

Many studies evidenced associations between lipids and ischemic stroke,^2,4^ demonstrating how these molecules are involved in the etiology and progression of AIS. Serum cholesterol is an independent predictor for long-term functional outcomes, and higher serum total cholesterol levels have been associated with better prognosis.^34^ Triglycerides have been significantly associated with the risk of stroke and carotid atherosclerosis,^35^ but the biological mechanisms by which they could affect the 3 month death or the survival of AIS patients need further investigations. Since triglycerides are hydrolyzed to fatty acids to furnish alternative energy sources during stress conditions, we can hypothesize a situation of energy failure, thus leading to an increase in demand for energy and an enhanced transition from aerobic to anaerobic glycolysis. As a consequence, levels of pyruvate and lactate may change, altering their role in providing substitute energy fuel and in metabolic pathways of neuroprotection where lactate is normally largely involved.^36^ Moreover, citrate and ketone body levels can change to restore energy homeostasis. It demonstrated a significant increase of serum ketone bodies in response to angioplasty-induced ischemia applied in patients with stable angina, hypothesizing that changes in the metabolism of ketone bodies could be related to the reperfusion oxidative stress.^37^

We reported associations of LDL estimated at *t*_2_ with 3 month death and 3 month poor functional outcome. It has been shown that AIS is associated with adverse distributions of LDL and HDL subclasses, and particularly, short-term mortality is linked to increased levels of small dense LDL particles (sdLDL).^38^ Our results also evidence the role of LDL and VLDL cholesterol, estimated at *t*_2_, in increasing the risk of symptomatic intracranial hemorrhage development at 24 h.

Moreover, statistically significant associations between HDL-related parameters and adverse outcomes could reflect a general inflammation condition that characterizes the post-stroke course. Inflammation may alter the lipoprotein profile by modulating the HDL function^39^ which contributes to the development of adverse outcomes, linked to the activity of rt-PA itself. Indeed, generated plasmin after rt-PA activity on plasminogen can degrade non-target proteins, including Apo A1, which represents the major protein constituent of HDL particles.

## Supporting Information Available

Complete list of metabolites and lipoproteins correctly assigned and quantified in serum NMR spectra; comparison between demographic and clinical characteristics of patients enrolled in the original study (*n* = 327) and in the presented metabolomic study (*n* = 243); Pearson correlation analysis performed to find the association between metabolites and lipids levels at pre (*t*_1_) rt-PA and inflammatory markers; Pearson correlation analysis performed to find the association between metabolite and lipid levels at pre (*t*_2_) rt-PA and inflammatory markers; complete list of the effects of pre (*t*_1_) rt-PA metabolites and lipids levels on early (i.e., sICH and non-response to intravenous thrombolysis intervention) and late (i.e., 3 month mortality and 3 month poor functional outcome) outcomes; complete list of the effects of 24 h post (*t*_2_) rt-PA metabolites and lipids levels on each evaluated outcome; and complete list of the effects of Δ(pre–post)_rt-PA_ metabolite and lipid levels on each evaluated outcome (PDF)

## Abbreviations

A2M: alpha2 macroglobulin
Apo: apolipoprotein
AUC: area under the curve
CRP: C reactive protein
HDL: high-density lipoprotein
ILs: interleukins
LDL: low-density lipoprotein
MAGIC: MArker bioloGici nell’Ictus Cerebrale
MCPI: monocyte chemo-attractant protein I
MMP9: metalloproteinase 9
mRS: modified Rankin scale
NIHSS: National Institutes of Health Stroke Scale
ROC: receiver operating characteristic
rT-PA: recombinant tissue plasminogen activator
SAP: serum amyloid protein
sICH: secondary intracerebral hemorrhage
TNFα: tumor necrosis factor alpha
VLDL: very low-density lipoprotein
