# Supplementary material for: Nuclear Magnetic Resonance-Based Metabolomics to Predict Early and Late Adverse Outcomes in Ischemic Stroke Treated with Intravenous Thrombolysis
Source: J Proteome Res. 2022 Dec 5;22(1):16–25. doi: 10.1021/acs.jproteome.2c00333 (PMC9830637; doi:10.1021/acs.jproteome.2c00333)
Supplement: Supplementary file 1 — pr2c00333_si_001.pdf [file pr2c00333_si_001.pdf]

NMR-BASED METABOLOMICS TO PREDICT EARLY AND LATE ADVERSE OUTCOMES IN ISCHEMIC STROKE TREATED WITH INTRAVENOUS THROMBOLYSIS

Cristina Licari[1]#, Leonardo Tenori[1,2]#, Francesca Di Cesare[1], Claudio Luchinat [1,2,3], Betti Giusti[4,5,6], Ada Kura[4,5], Rosina De Cario[4], Domenico Inzitari[7,8], Benedetta Piccardi[7], Mascia Nesi[7], Cristina Sarti[9], Francesco Arba[10], Vanessa Palumbo[7], PatriziaNencini[7], Rossella Marcucci[4,5,6], Anna Maria Gori[4,5,6], Elena Sticchi (Corresponding)[4]*

# contributed equally

Corresponding author email: elena.sticchi@unifi.it

[1] Magnetic Resonance Center (CERM), University of Florence, Via Luigi Sacconi 6, 50019, Sesto Fiorentino, Firenze, Italy 2

[2] Department of Chemistry “Ugo Schiff”, University of Florence, Via della Lastruccia 3–13, 50019, Sesto Fiorentino, Florence, Italy.

[3] C.I.R.M.M.P., Via Luigi Sacconi 6, 50019 Sesto Fiorentino, Florence, Italy

[4] Department of Experimental and Clinical Medicine, University of Florence, Largo Brambilla 3, Florence 50134, Italy.

[5]Atherothrombotic Diseases Center, Careggi Hospital, Florence, Largo Brambilla 3, Florence 50134, Italy.

[6] Excellence Centre for Research, Transfer and High Education for the Development of DE NOVO Therapies (DENOTHE), University of Florence, Viale Pieraccini 6, Firenze 50139, Italy.

[7] Stroke Unit, Careggi University Hospital, Florence 50134, Italy.

[8] Institute of Neuroscience, Italian National Research Council (CNR), Via Madonna del Piano, 10, Sesto Fiorentino, Florence 50019, Italy.

[9] NEUROFARBA Department, Neuroscience Section, University of Florence, Largo Brambilla 3, Florence 50134, Italy.

[10] Department of Neurology, Careggi University Hospital, Largo Brambilla 3, Florence 50134, Italy.

Supplementary Information

Table S1: Complete list of metabolites and lipoproteins correctly assigned and quantified in serum NMR spectra

**Metabolites**	**Lipoproteins**						
Creatinine	Trigl	LMF_Trigl_IDL	LMF_ApoB_IDL	SubPhosp_VLDL-2	SubFreeChol_LDL-3	SubTrigl_HDL-3	SubApoA2_HDL-1
Ala	Chol	LMF_Trigl_LDL	LMF_ApoB_LDL	SubPhosp_VLDL-3	SubFreeChol_LDL-4	SubTrigl_HDL-4	SubApoA2_HDL-2
Glu	LDL-Chol	LMF_Trigl_HDL	SubTrigl_VLDL-1	SubPhosp_VLDL-4	SubFreeChol_LDL-5	SubChol_HDL-1	SubApoA2_HDL-3
Gln	HDL-Chol	LMF_Chol_VLDL	SubTrigl_VLDL-2	SubPhosp_VLDL-5	SubFreeChol_LDL-6	SubChol_HDL-2	SubApoA2_HDL-4
Gly	Apo-A1	LMF_Chol_IDL	SubTrigl_VLDL-3	SubTrigl_LDL-1	SubPhosp_LDL-1	SubChol_HDL-3	
His	Apo-A2	LMF_Chol_LDL	SubTrigl_VLDL-4	SubTrigl_LDL-2	SubPhosp_LDL-2	SubChol_HDL-4	
Ile	Apo-B100	LMF_Chol_HDL	SubTrigl_VLDL-5	SubTrigl_LDL-3	SubPhosp_LDL-3	SubFreeChol_HDL-1	
Leu	Apo-B100-Apo-A1	LMF_FreeChol_VLDL	SubChol_VLDL-1	SubTrigl_LDL-4	SubPhosp_LDL-4	SubFreeChol_HDL-2	
Phe	VLDL_PN	LMF_FreeChol_IDL	SubChol_VLDL-2	SubTrigl_LDL-5	SubPhosp_LDL-5	SubFreeChol_HDL-3	
Tyr	IDL_PN	LMF_FreeChol_LDL	SubChol_VLDL-3	SubTrigl_LDL-6	SubPhosp_LDL-6	SubFreeChol_HDL-4	
Val	LDL_PN	LMF_FreeChol_HDL	SubChol_VLDL-4	SubChol_LDL-1	SubApoB_LDL-1	SubPhosp_HDL-1	
Acetic acid	LDL1_PN	LMF_Phosp_VLDL	SubChol_VLDL-5	SubChol_LDL-2	SubApoB_LDL-2	SubPhosp_HDL-2	
Citric acid	LDL2_PN	LMF_Phosp_IDL	SubFreeChol_VLDL-1	SubChol_LDL-3	SubApoB_LDL-3	SubPhosp_HDL-3	
Lactic acid	LDL3_PN	LMF_Phosp_LDL	SubFreeChol_VLDL-2	SubChol_LDL-4	SubApoB_LDL-4	SubPhosp_HDL-4	
3-HB	LDL4_PN	LMF_Phosp_HDL	SubFreeChol_VLDL-3	SubChol_LDL-5	SubApoB_LDL-5	SubApoA1_HDL-1	
Acetone	LDL5_PN	LMF_ApoA1_HDL	SubFreeChol_VLDL-4	SubChol_LDL-6	SubApoB_LDL-5	SubApoA1_HDL-2	
Pyruvic acid	LDL6_PN	LMF_ApoA2_HDL	SubFreeChol_VLDL-5	SubFreeChol_LDL-1	SubTrigl_HDL-1	SubApoA1_HDL-3	
Glucose	LMF_Trigl_VLDL	LMF_ApoB_VLDL	SubPhosp_VLDL-1	SubFreeChol_LDL-2	SubTrigl_HDL-2	SubApoA1_HDL-4	

*Main abbreviations: trigl: triglycerides; chol: cholesterol; phosp: phospholipids; Apo: apolipoprotein; LMF: lipoprotein main fraction; PN: particle number; sub: subfractions; phosp: phospholipids; 3-HB: 3-hydroxybutyrate. Amino acids are reported with the three letters code.

Table S2: Comparison between demographic and clinical characteristics of patients enrolled in the original study (*n* = 327) and the presented metabolomic study (*n* = 243).

**Demographics**	Patients cohort of the presented study (*n* = 243)	Patients cohort of the original study (*n* = 327)	*P*-value
Age, years, mean, and SD	68.8 ± 11.9	68.9 ± 12	0.88
Sex (male), n (%)	137/243 (56.4%)	190/327 (58.1%)	0.68
Onset to treatment time, minutes, mean, and SD	163.4 ± 83.7	163.5 ± 75.7	0.86
Baseline NIHSS, mean, and SD	11.9 ± 6.1	11.9 ± 6.0	0.94
Baseline systolic blood pressure, mmHg, mean, and SD	147.5 ± 21.3	148.2 ± 21.7	0.69
Baseline diastolic blood pressure, mmHg, mean, and SD	79.7 ± 12.7	80.1 ± 12.7	0.71
Blood glucose, mg/dL, mean, and SD	130.2 ± 49.5	130.2 ± 47.9	0.99
**Risk factors**			
Hypertension, n (%)	143/243 (58.8%)	197/327 (61.0%)	0.77
Diabetes, n (%)	36/243 (14.8%)	50/327 (15.4%)	0.89
Hyperlipidemia, n (%)	56/243 (23%)	81/327 (25.8%)	0.68
Current smoking, n (%)	35/243 (14.4%)	51/327 (15.9%)	0.70
Atrial fibrillation, n (%)	56/243 (23%)	73/327 (22.7%)	0.83
Congestive heart failure, n (%)	26/243 (10.7%)	35/327 (10.9%)	0.98

Abbreviation used: SD = Standard Deviation; NIHSS = National Institute of Health Stroke Scale

Table S3: Pearson correlation analysis bewteen molecular features and inflammatory markers evalutated in t_1. Only stastistically significant correlation were reported (*P*-value<0.05). For completeness FDR *P*-value was also reported

**Molecular features**	**Inflammatory markers**	***P*-value**	**FDR *P*-value**	**Correlation coefficient**
SubApoA2_HDL.4	CRP	2.82E-05	9.43E-05	0.265
SubApoA1_HDL.4	CRP	6.85E-05	0.0002	0.253
SubChol_HDL.4	CRP	0.0001	0.0004	0.244
SubFreeChol_HDL.4	CRP	0.0002	0.0007	0.235
SubPhosp_HDL.4	CRP	0.0003	0.001	0.228
SubApoA2_HDL.4	IL6	0.003	0.007	0.191
SubFreeChol_HDL.4	IL8	0.003	0.008	0.189
SubPhosp_HDL.4	IL6	0.004	0.009	0.185
SubChol_HDL.4	IL6	0.004	0.010	0.183
SubPhosp_HDL.4	IL8	0.008	0.019	0.169
SubChol_HDL.4	IL8	0.010	0.023	0.165
SubPhosp_LDL.4	CRP	0.012	0.027	0.162
LDL4_PN	CRP	0.012	0.028	0.160
SubApoB_LDL.4	CRP	0.012	0.028	0.160
SubTrigl_VLDL.3	IL12	0.013	0.028	0.160
SubChol_LDL.4	CRP	0.014	0.030	0.158
SubApoA1_HDL.4	IL6	0.014	0.031	0.158
LMF_FreeChol_LDL	CRP	0.016	0.036	0.154
Creatinine	MCPI	0.017	0.036	0.153
SubFreeChol_HDL.3	CRP	0.018	0.039	0.152
Glucose	TNFalpha	0.019	0.041	0.150
SubFreeChol_LDL.4	CRP	0.019	0.042	0.150
Apo.A2	CRP	0.022	0.046	0.147
SubFreeChol_LDL.5	CRP	0.023	0.049	0.146
SubTrigl_VLDL.4	IL12	0.024	0.051	0.145
SubChol_LDL.3	CRP	0.024	0.051	0.145
SubFreeChol_HDL.4	IL6	0.030	0.061	0.140
Glucose	IL8	0.030	0.063	0.139
SubTrigl_HDL.4	CRP	0.034	0.070	0.136
SubFreeChol_LDL.3	CRP	0.035	0.072	0.135
SubPhosp_VLDL.3	IL12	0.037	0.074	0.134
SubApoA2_HDL.4	IL8	0.037	0.074	0.134
SubFreeChol_HDL.4	IL10	0.039	0.078	0.133
SubPhosp_LDL.3	CRP	0.039	0.079	0.132
SubPhosp_VLDL.2	IL12	0.043	0.086	0.130
LMF_ApoA2_HDL	CRP	0.044	0.086	0.129
SubPhosp_LDL.5	CRP	0.047	0.093	0.127
SubApoA2_HDL.4	MCPI	0.048	0.093	0.127
SubTrigl_LDL.4	IL10	0.048	0.094	0.127
SubChol_HDL.3	IL8	0.048	0.095	-0.127
LMF_FreeChol_HDL	A2M	0.047	0.091	-0.128
LMF_FreeChol_HDL	A2M.1	0.047	0.091	-0.128
Val	IL10	0.046	0.091	-0.128
His	IL8	0.046	0.090	-0.128

Apo.A1	IL10	0.045	0.089	-0.129
SubTrigl_HDL.2	TNFalpha	0.044	0.087	-0.129
SubApoA1_HDL.2	IL12	0.044	0.086	-0.129
SubApoA1_HDL.1	IL1ra	0.043	0.084	-0.130
Val	TNFalpha	0.042	0.084	-0.130
SubChol_VLDL.1	IL10	0.042	0.084	-0.130
SubChol_HDL.3	IL10	0.041	0.082	-0.131
LMF_Phosp_HDL	MCPI	0.041	0.082	-0.131
SubPhosp_LDL.6	IL10	0.039	0.078	-0.132
SubChol_HDL.1	MMP9	0.039	0.078	-0.133
SubApoA2_HDL.1	IL12	0.038	0.076	-0.133
SubApoA1_HDL.3	IL12	0.038	0.076	-0.133
SubTrigl_VLDL.5	IL8	0.037	0.075	-0.134
Glu	IL8	0.037	0.074	-0.134
SubTrigl_LDL.6	IL10	0.037	0.074	-0.134
SubFreeChol_LDL.2	IL8	0.036	0.073	-0.135
Apo.A1	IL8	0.035	0.071	-0.135
SubFreeChol_LDL.1	IL8	0.034	0.069	-0.136
SubPhosp_HDL.1	IL12	0.034	0.069	-0.136
Gly	IL1ra	0.034	0.069	-0.136
Pyruvicacid	IL10	0.034	0.069	-0.136
SubFreeChol_HDL.1	IL12	0.033	0.067	-0.137
Glu	IL10	0.031	0.064	-0.138
Gly	IL10	0.031	0.063	-0.139
SubChol_HDL.2	IL1ra	0.030	0.063	-0.139
SubChol_VLDL.5	MCPI	0.030	0.062	-0.139
SubFreeChol_VLDL.5	IL10	0.030	0.062	-0.139
Val	IL12	0.029	0.060	-0.140
SubTrigl_HDL.1	MCPI	0.029	0.060	-0.140
Pyruvicacid	IL1ra	0.029	0.060	-0.140
Glu	IL1ra	0.028	0.059	-0.141
Ala	IL12	0.027	0.057	-0.142
SubTrigl_HDL.1	IL1ra	0.026	0.055	-0.143
SubApoB_LDL.6	IL10	0.026	0.054	-0.143
LDL6_PN	IL10	0.026	0.054	-0.143
SubTrigl_HDL.1	IL6	0.024	0.051	-0.145
LMF_Phosp_HDL	IL8	0.024	0.050	-0.145
SubChol_HDL.1	MCPI	0.023	0.050	-0.145
SubPhosp_VLDL.4	IL6	0.023	0.050	-0.145
LMF_Phosp_HDL	IL10	0.023	0.049	-0.146
SubChol_HDL.2	CRP	0.023	0.049	-0.146
SubPhosp_HDL.2	IL6	0.022	0.047	-0.147
LMF_ApoA1_HDL	IL10	0.022	0.047	-0.147
Pyruvicacid	MCPI	0.020	0.044	-0.149
SubPhosp_HDL.2	IL1ra	0.020	0.043	-0.149
Phe	TNFalpha	0.020	0.042	-0.150
Gly	CRP	0.020	0.042	-0.150

LMF_FreeChol_HDL	MCPI	0.019	0.042	-0.150
LMF_FreeChol_HDL	IL8	0.019	0.042	-0.150
LMF_FreeChol_IDL	IL6	0.019	0.041	-0.150
SubTrigl_VLDL.5	IL10	0.019	0.040	-0.151
Val	CRP	0.018	0.039	-0.152
SubApoA1_HDL.1	IL12	0.018	0.039	-0.152
SubTrigl_VLDL.5	IL1ra	0.017	0.037	-0.153
Ile	TNFalpha	0.017	0.037	-0.153
SubTrigl_HDL.3	IL1ra	0.017	0.037	-0.153
SubFreeChol_HDL.2	IL12	0.016	0.036	-0.154
SubFreeChol_HDL.2	IL10	0.016	0.035	-0.155
LMF_ApoA1_HDL	IL8	0.016	0.035	-0.155
SubPhosp_HDL.1	MCPI	0.016	0.035	-0.155
Lactic acid	MCPI	0.016	0.035	-0.155
Glu	IL12	0.016	0.034	-0.155
SubPhosp_LDL.1	IL8	0.016	0.034	-0.155
LMF_Trigl_HDL	IL1ra	0.015	0.033	-0.156
SubPhosp_HDL.2	CRP	0.015	0.033	-0.156
SubFreeChol_HDL.2	IL8	0.015	0.033	-0.156
SubChol_VLDL.1	IL8	0.015	0.032	-0.156
Ile	IL10	0.014	0.032	-0.157
SubTrigl_HDL.2	MCPI	0.014	0.032	-0.157
Ile	IL1ra	0.014	0.032	-0.157
Phe	IL6	0.014	0.031	-0.157
Val	IL8	0.014	0.031	-0.158
LMF_ApoB_IDL	IL6	0.013	0.030	-0.158
Ile	IL8	0.013	0.030	-0.159
IDL_PN	IL6	0.013	0.030	-0.159
His	IL10	0.013	0.030	-0.159
SubPhosp_VLDL.5	MCPI	0.013	0.030	-0.159
LMF_Chol_IDL	IL6	0.013	0.030	-0.159
SubFreeChol_HDL.1	MMP9	0.013	0.029	-0.160
SubChol_LDL.1	IL8	0.013	0.029	-0.160
SubApoA1_HDL.1	MCPI	0.012	0.028	-0.160
LMF_FreeChol_HDL	IL12	0.012	0.028	-0.160
SubPhosp_VLDL.5	CRP	0.012	0.028	-0.160
LMF_Trigl_HDL	IL6	0.012	0.027	-0.161
HDL.Chol	IL8	0.012	0.027	-0.161
LMF_Chol_HDL	IL8	0.012	0.027	-0.161
His	IL12	0.012	0.027	-0.162
HDL.Chol	IL10	0.012	0.027	-0.162
LMF_Chol_HDL	IL10	0.012	0.027	-0.162
Lactic acid	TNFalpha	0.011	0.026	-0.162
SubPhosp_VLDL.5	IL1ra	0.010	0.023	-0.165
SubTrigl_HDL.3	CRP	0.010	0.023	-0.165
Phe	IL1ra	0.009	0.022	-0.166
His	IL1ra	0.009	0.021	-0.168

SubTrigl_HDL.3	IL10	0.009	0.020	-0.168
SubChol_HDL.2	IL6	0.008	0.019	-0.169
SubFreeChol_HDL.1	IL8	0.008	0.019	-0.169
SubTrigl_HDL.2	IL12	0.008	0.019	-0.170
SubApoA2_HDL.2	IL10	0.008	0.019	-0.170
SubFreeChol_VLDL.5	IL8	0.008	0.018	-0.171
LMF_Trigl_HDL	IL10	0.008	0.018	-0.171
Gly	IL6	0.008	0.018	-0.171
SubPhosp_VLDL.5	IL6	0.008	0.018	-0.171
LDL1_PN	IL8	0.007	0.018	-0.171
SubApoB_LDL.1	IL8	0.007	0.018	-0.171
Lactic acid	IL1ra	0.007	0.018	-0.171
SubChol_VLDL.5	CRP	0.007	0.017	-0.172
SubApoA1_HDL.3	IL10	0.007	0.016	-0.173
Glu	MCPI	0.007	0.016	-0.173
TPN	IL6	0.007	0.016	-0.174
Apo.B100	IL6	0.007	0.016	-0.174
Tyr	IL12	0.006	0.014	-0.176
Leu	IL6	0.006	0.014	-0.177
Ile	IL12	0.006	0.014	-0.177
LMF_FreeChol_HDL	MMP9	0.005	0.013	-0.178
SubTrigl_VLDL.5	MCPI	0.005	0.013	-0.178
SubTrigl_LDL.2	IL6	0.005	0.012	-0.179
Leu	CRP	0.005	0.012	-0.179
SubApoA1_HDL.3	IL8	0.005	0.012	-0.180
SubTrigl_LDL.3	MCPI	0.005	0.012	-0.180
SubChol_VLDL.5	IL6	0.005	0.012	-0.180
Citricacid	CRP	0.005	0.012	-0.180
SubApoA2_HDL.2	IL8	0.005	0.012	-0.181
SubApoA1_HDL.2	IL8	0.005	0.011	-0.181
SubChol_VLDL.4	IL6	0.004	0.011	-0.182
Leu	TNFalpha	0.004	0.011	-0.182
SubChol_HDL.2	MCPI	0.004	0.010	-0.184
LMF_FreeChol_HDL	IL10	0.004	0.010	-0.184
Leu	MCPI	0.004	0.010	-0.184
SubFreeChol_VLDL.4	IL6	0.004	0.009	-0.186
SubTrigl_HDL.3	IL8	0.003	0.008	-0.189
SubTrigl_LDL.1	IL6	0.003	0.007	-0.190
LMF_Trigl_LDL	IL6	0.003	0.007	-0.190
SubPhosp_HDL.2	MCPI	0.003	0.007	-0.190
SubTrigl_HDL.2	IL1ra	0.003	0.007	-0.191
SubChol_HDL.1	IL10	0.003	0.007	-0.192
SubFreeChol_HDL.1	IL10	0.003	0.007	-0.192
SubTrigl_HDL.1	IL10	0.003	0.007	-0.192
SubApoA1_HDL.2	IL10	0.003	0.007	-0.192
SubApoA2_HDL.1	IL8	0.003	0.007	-0.192
Phe	IL10	0.002	0.006	-0.194

SubTrigl_LDL.3	IL6	0.002	0.006	-0.196
Apo.B100.Apo.A1	IL6	0.002	0.005	-0.198
SubPhosp_HDL.1	IL10	0.002	0.005	-0.199
SubApoA2_HDL.1	IL10	0.002	0.005	-0.199
SubChol_HDL.2	IL12	0.002	0.005	-0.200
SubTrigl_HDL.2	IL10	0.001	0.004	-0.204
Gly	IL8	0.001	0.004	-0.204
SubChol_HDL.1	IL8	0.001	0.004	-0.204
SubTrigl_VLDL.5	IL6	0.001	0.004	-0.205
SubTrigl_HDL.2	CRP	0.001	0.003	-0.205
Leu	IL8	0.001	0.003	-0.208
SubTrigl_VLDL.5	CRP	0.001	0.003	-0.208
Lactic acid	IL10	0.001	0.003	-0.208
Leu	IL1ra	0.001	0.003	-0.208
SubPhosp_HDL.2	IL12	0.001	0.003	-0.209
Gly	IL12	0.0010	0.003	-0.211
LMF_Trigl_HDL	IL8	0.0009	0.003	-0.211
SubPhosp_HDL.1	IL8	0.0009	0.003	-0.211
SubChol_LDL.1	IL6	0.0009	0.002	-0.212
SubApoA1_HDL.1	IL10	0.0008	0.002	-0.214
SubChol_LDL.2	IL6	0.0006	0.002	-0.218
Pyruvicacid	IL8	0.0005	0.002	-0.221
SubPhosp_LDL.2	IL6	0.0005	0.001	-0.223
SubPhosp_LDL.1	IL6	0.0004	0.001	-0.224
SubFreeChol_LDL.1	IL6	0.0004	0.001	-0.224
Gly	TNFalpha	0.0003	0.0008	-0.231
Leu	IL10	0.0003	0.0008	-0.231
SubChol_HDL.2	IL10	0.0003	0.0008	-0.233
SubPhosp_HDL.2	IL10	0.0002	0.0007	-0.235
SubApoA1_HDL.1	IL8	0.0002	0.0006	-0.236
SubTrigl_HDL.1	IL8	0.0002	0.0005	-0.239
SubApoB_LDL.1	IL6	0.0002	0.0005	-0.239
LDL1_PN	IL6	0.0002	0.0005	-0.239
SubTrigl_HDL.2	IL8	0.0002	0.0005	-0.240
LDL2_PN	IL6	0.0001	0.0003	-0.246
SubApoB_LDL.2	IL6	0.0001	0.0003	-0.246
SubTrigl_HDL.3	IL6	6.31E-05	0.0002	-0.254
SubFreeChol_LDL.2	IL6	5.89E-05	0.0002	-0.255
Leu	IL12	5.49E-05	0.0002	-0.256
Phe	IL12	5.22E-05	0.0002	-0.256
SubPhosp_HDL.2	IL8	3.15E-05	0.0001	-0.264
SubChol_HDL.2	IL8	2.55E-05	8.57E-05	-0.267
SubTrigl_HDL.2	IL6	1.54E-05	5.32E-05	-0.273
Phe	CRP	1.52E-05	5.23E-05	-0.274
Lactic acid	IL8	4.67E-10	2.29E-09	-0.386

Table S4: Pearson correlation analysis bewteen molecular features and inflammatory markers evalutated in t_2. Only stastistically significant correlation were reported (*P*-value<0.05). For completeness FDR *P*-value was also reported

**Molecular features**	**Metalloproteins**	***P*-value**	**FDR *P*-value**	**Correlation coefficient**
SubTrigl_HDL.2	CRP	2.46E-07	9.45E-07	-0.324
SubTrigl_HDL.3	CRP	9.19E-07	3.36E-06	-0.309
SubTrigl_LDL.1	IL6	2.65E-06	9.31E-06	-0.296
SubTrigl_HDL.1	CRP	3.55E-06	1.23E-05	-0.292
LMF_Trigl_HDL	CRP	4.75E-06	1.63E-05	-0.289
SubTrigl_HDL.3	IL6	2.97E-05	9.47E-05	-0.264
SubTrigl_LDL.2	IL6	4.67E-05	0.0001	-0.258
Gly	CRP	6.05E-05	0.0002	-0.254
LMF_Trigl_LDL	IL6	6.11E-05	0.0002	-0.254
SubApoB_LDL.1	IL6	0.0001	0.0003	-0.245
LDL1_PN	IL6	0.0001	0.0003	-0.245
SubPhosp_LDL.1	IL6	0.0002	0.0005	-0.240
SubTrigl_HDL.2	IL6	0.0004	0.001	-0.225
LMF_Trigl_HDL	IL6	0.0004	0.001	-0.224
SubTrigl_LDL.3	IL6	0.0006	0.002	-0.218
SubChol_LDL.1	IL6	0.0007	0.002	-0.217
SubFreeChol_LDL.1	IL6	0.0007	0.002	-0.216
IDL_PN	CRP	0.001	0.003	-0.208
LMF_ApoB_IDL	CRP	0.001	0.003	-0.208
IDL_PN	IL6	0.001	0.003	-0.208
LMF_ApoB_IDL	IL6	0.001	0.003	-0.208
SubFreeChol_VLDL.5	CRP	0.002	0.004	-0.202
SubTrigl_HDL.4	IL8	0.002	0.004	-0.200
Gly	IL6	0.002	0.005	-0.199
SubApoA1_HDL.2	IL10	0.002	0.005	-0.199
SubTrigl_LDL.1	CRP	0.002	0.005	-0.198
Apo.A1	IL8	0.002	0.005	-0.196
LMF_Phosp_HDL	IL10	0.002	0.006	-0.194
LMF_ApoA1_HDL	IL8	0.003	0.006	-0.192
SubPhosp_HDL.3	IL10	0.003	0.007	-0.191
LMF_FreeChol_IDL	IL6	0.003	0.008	-0.189
SubTrigl_HDL.1	IL6	0.003	0.008	-0.187
SubPhosp_HDL.3	MCPI	0.004	0.010	-0.183
SubChol_VLDL.4	IL6	0.004	0.011	-0.182
SubApoA1_HDL.3	IL8	0.005	0.011	-0.182
SubFreeChol_VLDL.4	CRP	0.005	0.011	-0.181
SubPhosp_HDL.2	IL10	0.005	0.012	-0.179
LDL2_PN	IL6	0.005	0.012	-0.178
SubApoB_LDL.2	IL6	0.005	0.012	-0.178
LMF_Chol_IDL	IL6	0.005	0.013	-0.178
SubFreeChol_VLDL.4	IL6	0.006	0.014	-0.176
Apo.B100.Apo.A1	IL6	0.006	0.015	-0.175
LMF_Phosp_HDL	MCPI	0.006	0.015	-0.175
Ile	CRP	0.006	0.015	-0.174

SubChol_VLDL.1	CRP	0.007	0.015	-0.174
LMF_FreeChol_IDL	CRP	0.007	0.016	-0.173
LMF_Chol_IDL	CRP	0.008	0.018	-0.171
Pyruvicacid	MCPI	0.008	0.018	-0.171
SubTrigl_LDL.3	MCPI	0.008	0.019	-0.169
SubChol_VLDL.5	IL6	0.010	0.022	-0.166
VLDL_PN	IL6	0.010	0.022	-0.166
LMF_ApoB_VLDL	IL6	0.010	0.022	-0.166
SubPhosp_HDL.3	IL8	0.010	0.022	-0.165
SubApoA2_HDL.2	CRP	0.010	0.023	-0.165
SubPhosp_LDL.1	MCPI	0.010	0.023	-0.165
SubTrigl_LDL.2	CRP	0.011	0.025	-0.162
SubPhosp_VLDL.4	IL6	0.011	0.025	-0.162
Glucose	MCPI	0.012	0.027	-0.160
SubChol_VLDL.5	IL1ra	0.013	0.028	-0.160
SubPhosp_HDL.2	MCPI	0.013	0.028	-0.160
LMF_Trigl_LDL	CRP	0.013	0.029	-0.159
SubTrigl_VLDL.5	CRP	0.014	0.030	-0.158
Trigl	CRP	0.014	0.031	-0.157
Gln	IL8	0.014	0.031	-0.157
SubTrigl_HDL.4	IL6	0.014	0.031	-0.157
SubFreeChol_VLDL.1	CRP	0.014	0.031	-0.157
LMF_ApoA1_HDL	IL10	0.016	0.034	-0.155
Apo.A1	IL10	0.016	0.035	-0.154
SubApoA1_HDL.2	MCPI	0.016	0.035	-0.154
SubPhosp_LDL.1	TNFalpha	0.017	0.037	-0.153
LMF_ApoB_VLDL	CRP	0.017	0.037	-0.152
VLDL_PN	CRP	0.017	0.037	-0.152
SubChol_VLDL.5	A2M	0.018	0.038	-0.152
SubChol_VLDL.5	A2M.1	0.018	0.038	-0.152
SubFreeChol_LDL.2	IL6	0.018	0.039	-0.151
SubApoB_LDL.1	MCPI	0.019	0.040	-0.151
LDL1_PN	MCPI	0.019	0.040	-0.150
SubPhosp_LDL.2	IL6	0.019	0.040	-0.150
SubChol_HDL.2	IL10	0.020	0.042	-0.149
SubApoA1_HDL.3	IL10	0.020	0.042	-0.149
SubChol_VLDL.5	TNFalpha	0.020	0.043	-0.149
SubPhosp_LDL.1	IL8	0.021	0.044	-0.148
HDL.Chol	MMP9	0.021	0.044	-0.148
LMF_Chol_HDL	MMP9	0.021	0.044	-0.148
LDL1_PN	CRP	0.021	0.045	-0.147
SubChol_LDL.1	MCPI	0.021	0.045	-0.147
SubApoB_LDL.1	CRP	0.021	0.045	-0.147
SubTrigl_VLDL.5	A2M	0.022	0.045	-0.147
SubTrigl_VLDL.5	A2M.1	0.022	0.045	-0.147
SubChol_LDL.1	IL8	0.022	0.045	-0.147
SubFreeChol_LDL.1	IL8	0.022	0.046	-0.147

HDL.Chol	IL10	0.022	0.046	-0.147
LMF_Chol_HDL	IL10	0.022	0.046	-0.147
SubPhosp_HDL.2	CRP	0.022	0.047	-0.146
Gln	IL6	0.023	0.047	-0.146
LMF_Chol_VLDL	CRP	0.023	0.047	-0.146
Val	CRP	0.023	0.048	-0.146
SubPhosp_VLDL.5	IL6	0.023	0.048	-0.145
SubPhosp_VLDL.1	CRP	0.023	0.049	-0.145
LMF_Chol_VLDL	IL6	0.024	0.049	-0.145
LDL1_PN	TNFalpha	0.024	0.050	-0.145
TPN	IL6	0.024	0.050	-0.145
Apo.B100	IL6	0.024	0.050	-0.145
SubApoB_LDL.1	TNFalpha	0.024	0.050	-0.145
SubChol_VLDL.5	IL12	0.025	0.051	-0.144
SubChol_LDL.1	TNFalpha	0.025	0.052	-0.144
SubApoA1_HDL.1	IL10	0.026	0.053	-0.143
SubPhosp_VLDL.5	IL8	0.027	0.055	-0.142
SubTrigl_LDL.2	MCPI	0.027	0.055	-0.142
SubApoA1_HDL.4	IL8	0.027	0.056	-0.141
SubTrigl_LDL.1	MCPI	0.028	0.056	-0.141
SubChol_VLDL.4	CRP	0.028	0.056	-0.141
SubTrigl_VLDL.5	IL6	0.029	0.058	-0.140
SubChol_HDL.2	MCPI	0.030	0.061	-0.139
SubTrigl_HDL.4	IL1ra	0.030	0.061	-0.139
SubChol_VLDL.5	IL8	0.030	0.061	-0.139
Gln	MCPI	0.031	0.061	-0.139
SubFreeChol_VLDL.5	IL6	0.031	0.062	-0.139
SubChol_LDL.2	IL6	0.031	0.063	-0.138
LMF_Trigl_HDL	IL8	0.033	0.065	-0.137
SubChol_VLDL.5	MCPI	0.033	0.066	-0.137
SubPhosp_HDL.1	A2M	0.033	0.066	-0.137
SubPhosp_HDL.1	A2M.1	0.033	0.066	-0.137
SubFreeChol_LDL.1	TNFalpha	0.034	0.067	-0.136
SubChol_VLDL.2	IL6	0.034	0.068	-0.136
SubTrigl_HDL.1	A2M	0.034	0.068	-0.136
SubTrigl_HDL.1	A2M.1	0.034	0.068	-0.136
SubTrigl_LDL.2	TNFalpha	0.036	0.071	-0.135
SubTrigl_LDL.1	IL1ra	0.036	0.071	-0.134
SubApoA1_HDL.1	A2M	0.036	0.071	-0.134
SubApoA1_HDL.1	A2M.1	0.036	0.071	-0.134
LMF_Phosp_IDL	IL6	0.038	0.075	-0.133
SubPhosp_VLDL.4	CRP	0.039	0.076	-0.133
SubFreeChol_LDL.1	MCPI	0.039	0.076	-0.132
SubPhosp_VLDL.5	IL1ra	0.040	0.078	-0.132
LMF_Phosp_HDL	IL8	0.040	0.078	-0.132
Gly	TNFalpha	0.041	0.079	-0.131
SubTrigl_VLDL.1	CRP	0.041	0.079	-0.131

Leu	CRP	0.041	0.080	-0.131
SubChol_HDL.3	IL8	0.042	0.081	-0.131
SubApoB_LDL.1	IL8	0.042	0.082	-0.130
LDL1_PN	IL8	0.042	0.082	-0.130
SubPhosp_LDL.1	CRP	0.044	0.084	-0.129
Gln	IL1ra	0.044	0.085	-0.129
LMF_ApoA1_HDL	MMP9	0.044	0.085	-0.129
SubTrigl_LDL.5	IL6	0.045	0.086	-0.129
Trigl	IL6	0.045	0.086	-0.129
SubTrigl_LDL.4	IL6	0.045	0.087	-0.129
SubFreeChol_VLDL.3	CRP	0.045	0.087	-0.128
SubApoA1_HDL.1	CRP	0.046	0.088	-0.128
LMF_ApoA1_HDL	MCPI	0.046	0.089	-0.128
SubChol_HDL.1	A2M	0.047	0.089	-0.128
SubChol_HDL.1	A2M.1	0.047	0.089	-0.128
His	IL10	0.048	0.092	-0.127
SubFreeChol_LDL.6	IL12	0.050	0.094	0.126
SubFreeChol_VLDL.2	IL12	0.048	0.091	0.127
SubFreeChol_LDL.6	IL1ra	0.044	0.085	0.129
Glu	TNFalpha	0.041	0.080	0.131
SubChol_LDL.6	TNFalpha	0.041	0.079	0.131
SubTrigl_VLDL.3	IL12	0.041	0.079	0.131
SubFreeChol_VLDL.4	IL12	0.039	0.076	0.132
LMF_FreeChol_IDL	IL12	0.037	0.072	0.134
SubChol_VLDL.4	IL12	0.034	0.067	0.136
SubPhosp_VLDL.2	IL12	0.034	0.067	0.136
SubChol_LDL.6	IL12	0.033	0.066	0.137
Lactic acid	IL1ra	0.030	0.061	0.139
Citricacid	IL8	0.028	0.057	0.141
LDL4_PN	CRP	0.024	0.050	0.144
SubApoB_LDL.4	CRP	0.024	0.050	0.144
LMF_Chol_IDL	IL12	0.024	0.049	0.145
SubApoA2_HDL.4	IL6	0.022	0.046	0.147
Citricacid	IL12	0.022	0.045	0.147
Lactic acid	MCPI	0.020	0.042	0.149
Citricacid	IL10	0.019	0.040	0.150
SubFreeChol_HDL.4	IL6	0.019	0.040	0.151
Glucose	MMP9	0.019	0.039	0.151
SubFreeChol_VLDL.3	IL12	0.018	0.039	0.151
X3.HB	IL10	0.017	0.036	0.153
LDL3_PN	CRP	0.016	0.035	0.154
SubApoB_LDL.3	CRP	0.016	0.035	0.154
Phe	IL8	0.016	0.035	0.154
Lactic acid	IL8	0.015	0.032	0.156
SubTrigl_LDL.6	IL1ra	0.015	0.032	0.156
SubChol_HDL.4	IL6	0.014	0.031	0.157
SubChol_VLDL.3	IL12	0.011	0.025	0.163

SubPhosp_VLDL.3	IL12	0.009	0.020	0.168
SubChol_LDL.4	CRP	0.008	0.018	0.170
SubPhosp_LDL.4	CRP	0.008	0.018	0.171
Val	MMP9	0.008	0.018	0.171
Tyr	IL8	0.007	0.017	0.172
SubFreeChol_LDL.4	CRP	0.007	0.016	0.174
SubFreeChol_HDL.3	CRP	0.007	0.015	0.174
SubPhosp_LDL.3	CRP	0.003	0.008	0.187
SubFreeChol_LDL.3	CRP	0.002	0.005	0.199
SubApoA1_HDL.4	CRP	0.002	0.005	0.199
Lactic acid	IL12	0.002	0.005	0.199
SubChol_LDL.3	CRP	0.001	0.004	0.203
SubApoA2_HDL.4	CRP	0.0006	0.002	0.218
SubPhosp_HDL.4	CRP	0.0004	0.001	0.225
SubChol_HDL.4	CRP	6.43E-05	0.0002	0.253
SubFreeChol_HDL.4	CRP	3.10E-05	9.86E-05	0.264

Table S5: Effect of pre (t_1) rt-PA metabolites and lipids levels on early (*i.e.* sICH, non-response to thrombolysis) and late (*i.e.* three-month mortality and three-month mRS 3-6) adverse outcomes, adjusting for the major determinants for unfavourable outcomes.

	Early outcomes						Late outcomes					
	sICH			Non-response to thrombolysis			Three-month mortality			Three-month mRS 3-6		
**Molecular features**	**OR (95% CI)**	**P**	**FDR**	**OR (95% CI)**	**P**	**FDR**	**OR (95% CI)**	**P**	**FDR**	**OR (95% CI)**	**P**	**FDR**
Creatinine	1.072 (0.763-1.506)	0.725	0.998	1.272 (0.932-1.737)	0.102	0.615	1.512 (1.042-2.193)	0.100	0.777	0.908 (0.633-1.302)	0.619	0.880
Ala	0.844 (0.511-1.393)	0.549	0.998	0.935 (0.699-1.25)	0.650	0.909	1.144 (0.754-1.734)	0.562	0.869	1.235 (0.865-1.765)	0.262	0.880
Glu	0.955 (0.606-1.507)	0.860	0.998	1.086 (0.818-1.443)	0.570	0.909	1.263 (0.844-1.89)	0.300	0.869	0.837 (0.586-1.196)	0.334	0.880
Gln	1.188 (0.707-1.996)	0.557	0.998	0.931 (0.684-1.267)	0.650	0.909	1.043 (0.627-1.736)	0.884	0.936	0.879 (0.617-1.251)	0.484	0.880
Gly	0.819 (0.487-1.378)	0.489	0.998	0.906 (0.679-1.21)	0.507	0.909	1.158 (0.747-1.794)	0.549	0.869	1.047 (0.734-1.495)	0.806	0.931
His	1.075 (0.827-1.396)	0.635	0.998	0.92 (0.748-1.131)	0.424	0.909	1.149 (0.918-1.439)	0.220	0.793	1.103 (0.891-1.365)	0.367	0.880
Ile	0.985 (0.632-1.535)	0.951	0.998	0.981 (0.75-1.282)	0.886	0.940	1.003 (0.66-1.524)	0.990	0.990	1.004 (0.711-1.417)	0.984	0.984
Leu	0.977 (0.635-1.503)	0.925	0.998	0.948 (0.728-1.236)	0.697	0.909	1.144 (0.772-1.695)	0.552	0.869	1.087 (0.785-1.506)	0.636	0.880
Phe	0.977 (0.636-1.502)	0.925	0.998	0.877 (0.669-1.148)	0.341	0.909	1.095 (0.742-1.615)	0.676	0.869	1.232 (0.897-1.69)	0.216	0.880
Tyr	0.883 (0.561-1.392)	0.626	0.998	0.947 (0.715-1.255)	0.707	0.909	1.137 (0.748-1.728)	0.578	0.869	1.296 (0.92-1.826)	0.152	0.880
Val	0.737 (0.454-1.194)	0.252	0.998	1.011 (0.76-1.346)	0.940	0.940	1.598 (0.989-2.58)	0.074	0.777	1.099 (0.781-1.547)	0.598	0.880
Acetic acid	1.001 (0.621-1.615)	0.998	0.998	1.142 (0.853-1.528)	0.354	0.909	1.09 (0.781-1.52)	0.673	0.869	0.976 (0.715-1.333)	0.882	0.934
Citric acid	1.291 (0.867-1.922)	0.450	0.998	1.14 (0.786-1.655)	0.498	0.909	1.075 (0.738-1.566)	0.737	0.884	0.938 (0.611-1.44)	0.828	0.931
Lactic acid	0.845 (0.453-1.578)	0.642	0.998	1.017 (0.763-1.357)	0.908	0.940	1.431 (0.898-2.281)	0.197	0.793	1.093 (0.769-1.555)	0.634	0.880
3-HB	1.457 (1.062-1.998)	0.025	0.455	0.718 (0.531-0.97)	0.024	0.219	0.897 (0.605-1.33)	0.604	0.869	1.121 (0.833-1.508)	0.465	0.880
Acetone	1.393 (1.016-1.909)	0.046	0.502	0.696 (0.509-0.95)	0.017	0.219	0.963 (0.634-1.465)	0.874	0.936	1.148 (0.836-1.576)	0.407	0.880
Pyruvic acid	1.124 (0.713-1.77)	0.738	0.998	1.09 (0.827-1.435)	0.537	0.909	1.456 (1.021-2.077)	0.129	0.777	1.077 (0.76-1.525)	0.723	0.929
Glucose	0.828 (0.343-1.998)	0.712	0.998	0.96 (0.679-1.357)	0.818	0.940	1.293 (0.564-2.965)	0.578	0.869	1.364 (0.721-2.58)	0.354	0.880
Trigl	1.053 (0.684-1.62)	0.832	0.999	1.131 (0.862-1.484)	0.375	0.956	0.839 (0.497-1.417)	0.549	0.994	1.09 (0.768-1.547)	0.641	0.812
Chol	0.864 (0.541-1.38)	0.578	0.999	0.96 (0.721-1.278)	0.780	0.998	0.947 (0.568-1.58)	0.851	0.994	1.207 (0.841-1.734)	0.320	0.629
LDL-Chol	0.933 (0.593-1.468)	0.787	0.999	0.916 (0.688-1.221)	0.551	0.966	1.034 (0.632-1.69)	0.905	0.994	1.377 (0.965-1.965)	0.083	0.392
HDL-Chol	0.997 (0.638-1.558)	0.991	0.999	0.908 (0.679-1.216)	0.521	0.956	0.746 (0.457-1.216)	0.273	0.994	0.741 (0.516-1.063)	0.111	0.448
Apo-A1	0.936 (0.591-1.483)	0.795	0.999	1.035 (0.767-1.396)	0.822	0.998	0.704 (0.428-1.157)	0.195	0.994	0.698 (0.478-1.018)	0.066	0.387

Apo-A2	0.854 (0.54-1.35)	0.535	0.999	0.946 (0.7-1.277)	0.717	0.998	0.861 (0.523-1.416)	0.589	0.994	0.825 (0.574-1.187)	0.311	0.623
Apo-B100	0.967 (0.609-1.536)	0.898	0.999	1.033 (0.783-1.362)	0.820	0.998	0.94 (0.556-1.589)	0.836	0.994	1.403 (0.982-2.004)	0.068	0.387
Apo-B100-Apo-A1	0.996 (0.663-1.497)	0.987	0.999	0.988 (0.751-1.299)	0.932	0.998	1.212 (0.803-1.831)	0.395	0.994	1.63 (1.131-2.349)	0.007	0.209
VLDL_PN	1.073 (0.708-1.626)	0.762	0.999	1.203 (0.915-1.581)	0.183	0.956	0.857 (0.521-1.411)	0.579	0.994	1.068 (0.752-1.516)	0.722	0.858
IDL_PN	1.006 (0.651-1.553)	0.981	0.999	1.14 (0.868-1.499)	0.347	0.956	1.009 (0.624-1.63)	0.975	0.994	1.144 (0.807-1.621)	0.465	0.680
LDL_PN	0.975 (0.622-1.529)	0.920	0.999	0.942 (0.713-1.244)	0.674	0.998	1.095 (0.664-1.806)	0.747	0.994	1.392 (0.974-1.989)	0.075	0.387
LDL1_PN	1.165 (0.745-1.823)	0.542	0.999	1 (0.745-1.344)	0.998	0.998	0.936 (0.572-1.533)	0.812	0.994	1.006 (0.705-1.434)	0.975	0.984
LDL2_PN	1.131 (0.721-1.775)	0.623	0.999	0.998 (0.74-1.346)	0.991	0.998	0.77 (0.465-1.275)	0.353	0.994	1.153 (0.822-1.618)	0.419	0.680
LDL3_PN	1.01 (0.643-1.587)	0.968	0.999	0.879 (0.658-1.175)	0.384	0.956	1.271 (0.774-2.088)	0.394	0.994	1.335 (0.934-1.907)	0.121	0.448
LDL4_PN	0.957 (0.638-1.436)	0.842	0.999	0.933 (0.704-1.238)	0.632	0.998	1.401 (0.924-2.124)	0.164	0.994	1.109 (0.799-1.541)	0.546	0.731
LDL5_PN	0.884 (0.557-1.401)	0.626	0.999	1.025 (0.773-1.359)	0.866	0.998	1.081 (0.654-1.784)	0.784	0.994	1.25 (0.885-1.764)	0.216	0.564
LDL6_PN	0.827 (0.492-1.389)	0.515	0.999	0.978 (0.745-1.285)	0.874	0.998	0.949 (0.533-1.689)	0.875	0.994	1.418 (1.022-1.967)	0.044	0.331
LMF_Trigl_VLDL	1.007 (0.643-1.578)	0.978	0.999	1.111 (0.845-1.461)	0.452	0.956	0.871 (0.515-1.475)	0.644	0.994	1.154 (0.811-1.641)	0.442	0.680
LMF_Trigl_IDL	1.09 (0.717-1.656)	0.717	0.999	1.125 (0.86-1.471)	0.391	0.956	0.825 (0.489-1.392)	0.508	0.994	1.047 (0.741-1.479)	0.801	0.897
LMF_Trigl_LDL	1.103 (0.741-1.641)	0.659	0.999	1.156 (0.876-1.524)	0.305	0.956	1.034 (0.671-1.594)	0.887	0.994	1.146 (0.814-1.614)	0.449	0.680
LMF_Trigl_HDL	1.06 (0.691-1.625)	0.806	0.999	1.375 (1.016-1.862)	0.036	0.956	0.711 (0.43-1.177)	0.220	0.994	0.791 (0.554-1.13)	0.204	0.564
LMF_Chol_VLDL	0.906 (0.575-1.428)	0.698	0.999	1.209 (0.92-1.587)	0.171	0.956	0.856 (0.497-1.473)	0.614	0.994	1.069 (0.754-1.516)	0.718	0.858
LMF_Chol_IDL	0.892 (0.56-1.42)	0.660	0.999	1.14 (0.865-1.502)	0.354	0.956	1.039 (0.62-1.742)	0.896	0.994	1.239 (0.869-1.765)	0.251	0.584
LMF_Chol_LDL	0.933 (0.593-1.468)	0.787	0.999	0.916 (0.688-1.221)	0.551	0.966	1.034 (0.632-1.69)	0.905	0.994	1.377 (0.965-1.965)	0.083	0.392
LMF_Chol_HDL	0.997 (0.638-1.558)	0.991	0.999	0.908 (0.679-1.216)	0.521	0.956	0.746 (0.457-1.216)	0.273	0.994	0.741 (0.516-1.063)	0.111	0.448
LMF_FreeChol_VLDL	0.964 (0.611-1.52)	0.885	0.999	1.166 (0.887-1.534)	0.271	0.956	0.796 (0.458-1.383)	0.462	0.994	1.065 (0.748-1.517)	0.735	0.864
LMF_FreeChol_IDL	0.93 (0.588-1.471)	0.778	0.999	1.147 (0.871-1.511)	0.329	0.956	0.991 (0.594-1.655)	0.976	0.994	1.192 (0.835-1.702)	0.347	0.671
LMF_FreeChol_LDL	0.873 (0.551-1.382)	0.596	0.999	0.895 (0.669-1.198)	0.458	0.956	1.189 (0.713-1.985)	0.551	0.994	1.363 (0.947-1.961)	0.101	0.445
LMF_FreeChol_HDL	0.763 (0.486-1.197)	0.275	0.999	0.901 (0.67-1.211)	0.491	0.956	0.865 (0.532-1.407)	0.592	0.994	0.872 (0.591-1.287)	0.506	0.714
LMF_Phosp_VLDL	1 (0.637-1.572)	0.999	0.999	1.131 (0.858-1.491)	0.383	0.956	0.852 (0.505-1.438)	0.586	0.994	1.04 (0.729-1.484)	0.832	0.912
LMF_Phosp_IDL	0.967 (0.608-1.538)	0.898	0.999	1.118 (0.85-1.47)	0.425	0.956	0.945 (0.565-1.581)	0.846	0.994	1.155 (0.812-1.643)	0.438	0.680
LMF_Phosp_LDL	0.974 (0.618-1.537)	0.920	0.999	0.905 (0.678-1.207)	0.497	0.956	1.083 (0.659-1.779)	0.776	0.994	1.303 (0.911-1.866)	0.156	0.528
LMF_Phosp_HDL	1.079 (0.676-1.723)	0.772	0.999	0.955 (0.711-1.283)	0.762	0.998	0.793 (0.477-1.319)	0.409	0.994	0.584 (0.393-0.867)	0.008	0.209
LMF_ApoA1_HDL	0.989 (0.633-1.544)	0.964	0.999	1.072 (0.796-1.444)	0.649	0.998	0.627 (0.379-1.038)	0.086	0.994	0.674 (0.465-0.978)	0.040	0.331
LMF_ApoA2_HDL	0.882 (0.556-1.398)	0.625	0.999	0.951 (0.706-1.28)	0.740	0.998	0.841 (0.509-1.389)	0.535	0.994	0.821 (0.571-1.18)	0.298	0.623
LMF_ApoB_VLDL	1.073 (0.708-1.626)	0.762	0.999	1.203 (0.915-1.581)	0.183	0.956	0.857 (0.521-1.412)	0.580	0.994	1.068 (0.752-1.516)	0.722	0.858
LMF_ApoB_IDL	1.006 (0.652-1.553)	0.981	0.999	1.141 (0.868-1.499)	0.346	0.956	1.009 (0.624-1.63)	0.975	0.994	1.144 (0.807-1.621)	0.465	0.680
LMF_ApoB_LDL	0.975 (0.622-1.529)	0.920	0.999	0.942 (0.714-1.244)	0.675	0.998	1.095 (0.664-1.806)	0.747	0.994	1.392 (0.974-1.989)	0.075	0.387
SubTrigl_VLDL-1	1.01 (0.642-1.59)	0.968	0.999	1.048 (0.794-1.383)	0.743	0.998	0.855 (0.508-1.441)	0.592	0.994	1.16 (0.811-1.659)	0.433	0.680
SubTrigl_VLDL-2	0.87 (0.553-1.37)	0.576	0.999	1.152 (0.873-1.52)	0.317	0.956	1.072 (0.647-1.777)	0.807	0.994	1.246 (0.881-1.763)	0.233	0.564
SubTrigl_VLDL-3	0.894 (0.565-1.415)	0.660	0.999	1.199 (0.906-1.588)	0.203	0.956	0.995 (0.593-1.668)	0.985	0.994	1.227 (0.858-1.755)	0.278	0.610
SubTrigl_VLDL-4	1.038 (0.672-1.602)	0.878	0.999	1.221 (0.925-1.613)	0.157	0.956	0.904 (0.551-1.484)	0.716	0.994	1.102 (0.777-1.562)	0.596	0.781
SubTrigl_VLDL-5	1.169 (0.764-1.79)	0.510	0.999	1.099 (0.839-1.439)	0.496	0.956	0.758 (0.473-1.215)	0.298	0.994	0.801 (0.568-1.131)	0.219	0.564
SubChol_VLDL-1	0.993 (0.632-1.56)	0.977	0.999	1.113 (0.843-1.469)	0.451	0.956	0.819 (0.477-1.407)	0.510	0.994	1.074 (0.751-1.535)	0.706	0.858
SubChol_VLDL-2	0.866 (0.552-1.356)	0.563	0.999	1.224 (0.932-1.608)	0.143	0.956	0.976 (0.577-1.65)	0.935	0.994	1.06 (0.75-1.498)	0.750	0.872
SubChol_VLDL-3	0.832 (0.52-1.332)	0.482	0.999	1.222 (0.927-1.611)	0.153	0.956	1 (0.588-1.702)	0.999	0.999	1.184 (0.835-1.679)	0.360	0.680
SubChol_VLDL-4	0.855 (0.54-1.353)	0.536	0.999	1.249 (0.946-1.65)	0.115	0.956	0.928 (0.554-1.554)	0.796	0.994	1.042 (0.735-1.476)	0.824	0.912
SubChol_VLDL-5	1.293 (0.84-1.99)	0.283	0.999	1.113 (0.84-1.475)	0.456	0.956	0.701 (0.431-1.141)	0.186	0.994	0.618 (0.427-0.893)	0.011	0.209
SubFreeChol_VLDL-1	1.04 (0.664-1.63)	0.876	0.999	1.11 (0.842-1.463)	0.460	0.956	0.806 (0.467-1.392)	0.479	0.994	1.074 (0.752-1.535)	0.704	0.858
SubFreeChol_VLDL-2	0.951 (0.611-1.481)	0.839	0.999	1.207 (0.92-1.583)	0.174	0.956	0.928 (0.547-1.577)	0.806	0.994	1.149 (0.811-1.628)	0.451	0.680
SubFreeChol_VLDL-3	0.942 (0.599-1.481)	0.813	0.999	1.188 (0.903-1.562)	0.217	0.956	0.922 (0.545-1.562)	0.787	0.994	1.176 (0.829-1.669)	0.380	0.680
SubFreeChol_VLDL-4	0.904 (0.574-1.424)	0.691	0.999	1.234 (0.938-1.623)	0.133	0.956	0.966 (0.577-1.62)	0.907	0.994	1.08 (0.764-1.528)	0.671	0.841

SubFreeChol_VLDL-5	1.201 (0.778-1.854)	0.457	0.999	1.101 (0.828-1.464)	0.508	0.956	0.673 (0.391-1.158)	0.177	0.994	0.816 (0.56-1.189)	0.303	0.623
SubPhosp_VLDL-1	0.983 (0.625-1.548)	0.948	0.999	1.066 (0.809-1.404)	0.653	0.998	0.832 (0.489-1.416)	0.537	0.994	1.16 (0.811-1.659)	0.431	0.680
SubPhosp_VLDL-2	0.873 (0.559-1.362)	0.578	0.999	1.177 (0.895-1.547)	0.243	0.956	1.055 (0.633-1.758)	0.851	0.994	1.223 (0.865-1.729)	0.274	0.610
SubPhosp_VLDL-3	0.937 (0.599-1.467)	0.795	0.999	1.229 (0.933-1.618)	0.140	0.956	0.971 (0.583-1.619)	0.920	0.994	1.178 (0.829-1.673)	0.375	0.680
SubPhosp_VLDL-4	0.964 (0.621-1.497)	0.881	0.999	1.262 (0.957-1.663)	0.097	0.956	0.951 (0.578-1.565)	0.856	0.994	1.058 (0.747-1.497)	0.758	0.873
SubPhosp_VLDL-5	1.164 (0.756-1.792)	0.527	0.999	1.147 (0.869-1.514)	0.333	0.956	0.67 (0.409-1.095)	0.142	0.994	0.641 (0.444-0.926)	0.019	0.268
SubTrigl_LDL-1	1.193 (0.821-1.734)	0.392	0.999	1.145 (0.869-1.51)	0.336	0.956	0.923 (0.602-1.415)	0.732	0.994	0.992 (0.704-1.399)	0.966	0.984
SubTrigl_LDL-2	1.252 (0.856-1.832)	0.285	0.999	1.116 (0.842-1.48)	0.445	0.956	1.018 (0.675-1.536)	0.937	0.994	1.024 (0.724-1.448)	0.896	0.946
SubTrigl_LDL-3	1.205 (0.809-1.794)	0.407	0.999	1.004 (0.762-1.323)	0.977	0.998	1.045 (0.681-1.605)	0.853	0.994	0.998 (0.707-1.409)	0.992	0.992
SubTrigl_LDL-4	1.054 (0.694-1.602)	0.820	0.999	1.096 (0.825-1.457)	0.526	0.956	1.286 (0.831-1.99)	0.300	0.994	1.048 (0.74-1.483)	0.799	0.897
SubTrigl_LDL-5	0.951 (0.616-1.469)	0.835	0.999	1.093 (0.834-1.432)	0.522	0.956	1.285 (0.807-2.048)	0.336	0.994	1.092 (0.778-1.532)	0.621	0.795
SubTrigl_LDL-6	0.792 (0.476-1.318)	0.412	0.999	0.917 (0.699-1.201)	0.528	0.956	1.224 (0.758-1.975)	0.465	0.994	1.464 (1.059-2.025)	0.024	0.268
SubChol_LDL-1	1.125 (0.709-1.786)	0.651	0.999	0.971 (0.722-1.306)	0.845	0.998	0.928 (0.563-1.531)	0.792	0.994	0.99 (0.697-1.407)	0.956	0.984
SubChol_LDL-2	1.101 (0.703-1.726)	0.699	0.999	0.96 (0.714-1.291)	0.790	0.998	0.765 (0.463-1.265)	0.340	0.994	1.163 (0.831-1.628)	0.389	0.680
SubChol_LDL-3	0.959 (0.618-1.489)	0.864	0.999	0.853 (0.64-1.138)	0.279	0.956	1.23 (0.758-1.994)	0.450	0.994	1.326 (0.934-1.883)	0.122	0.448
SubChol_LDL-4	0.917 (0.606-1.389)	0.699	0.999	0.892 (0.671-1.187)	0.431	0.956	1.406 (0.932-2.12)	0.153	0.994	1.122 (0.81-1.554)	0.499	0.714
SubChol_LDL-5	0.898 (0.568-1.417)	0.667	0.999	0.993 (0.746-1.321)	0.961	0.998	1.055 (0.639-1.743)	0.850	0.994	1.242 (0.877-1.757)	0.233	0.564
SubChol_LDL-6	0.826 (0.496-1.377)	0.505	0.999	0.935 (0.71-1.232)	0.634	0.998	0.96 (0.547-1.687)	0.900	0.994	1.461 (1.049-2.035)	0.029	0.280
SubFreeChol_LDL-1	1.032 (0.643-1.655)	0.906	0.999	1.001 (0.742-1.35)	0.993	0.998	0.926 (0.555-1.543)	0.788	0.994	1.033 (0.726-1.471)	0.859	0.925
SubFreeChol_LDL-2	1.013 (0.646-1.586)	0.960	0.999	1.004 (0.742-1.36)	0.977	0.998	0.695 (0.414-1.167)	0.202	0.994	1.162 (0.826-1.634)	0.399	0.680
SubFreeChol_LDL-3	0.987 (0.623-1.564)	0.959	0.999	0.867 (0.648-1.16)	0.338	0.956	1.142 (0.696-1.875)	0.635	0.994	1.264 (0.883-1.808)	0.210	0.564
SubFreeChol_LDL-4	0.953 (0.621-1.461)	0.837	0.999	0.873 (0.653-1.169)	0.361	0.956	1.312 (0.817-2.108)	0.325	0.994	1.228 (0.868-1.737)	0.258	0.588
SubFreeChol_LDL-5	0.883 (0.556-1.401)	0.624	0.999	0.964 (0.723-1.285)	0.802	0.998	1.109 (0.665-1.849)	0.721	0.994	1.29 (0.909-1.831)	0.162	0.528
SubFreeChol_LDL-6	0.741 (0.457-1.201)	0.257	0.999	0.93 (0.708-1.222)	0.605	0.998	1.016 (0.603-1.71)	0.958	0.994	1.575 (1.116-2.221)	0.011	0.209
SubPhosp_LDL-1	1.172 (0.74-1.855)	0.539	0.999	0.99 (0.735-1.333)	0.947	0.998	0.908 (0.55-1.499)	0.732	0.994	0.955 (0.669-1.362)	0.803	0.897
SubPhosp_LDL-2	1.136 (0.722-1.788)	0.612	0.999	0.973 (0.723-1.309)	0.859	0.998	0.786 (0.476-1.299)	0.392	0.994	1.122 (0.8-1.573)	0.513	0.714
SubPhosp_LDL-3	0.99 (0.634-1.545)	0.967	0.999	0.853 (0.638-1.14)	0.282	0.956	1.237 (0.759-2.015)	0.442	0.994	1.294 (0.909-1.843)	0.162	0.528
SubPhosp_LDL-4	0.946 (0.631-1.419)	0.801	0.999	0.883 (0.662-1.177)	0.392	0.956	1.418 (0.94-2.14)	0.142	0.994	1.135 (0.817-1.576)	0.462	0.680
SubPhosp_LDL-5	0.906 (0.576-1.426)	0.694	0.999	1 (0.752-1.33)	0.998	0.998	1.067 (0.649-1.753)	0.817	0.994	1.207 (0.853-1.709)	0.300	0.623
SubPhosp_LDL-6	0.836 (0.503-1.388)	0.531	0.999	0.929 (0.706-1.223)	0.601	0.998	0.956 (0.546-1.672)	0.888	0.994	1.392 (1.003-1.931)	0.056	0.379
SubApoB_LDL-1	1.165 (0.745-1.823)	0.541	0.999	1 (0.745-1.344)	0.998	0.998	0.936 (0.572-1.533)	0.813	0.994	1.006 (0.705-1.435)	0.975	0.984
SubApoB_LDL-2	1.132 (0.721-1.776)	0.623	0.999	0.998 (0.74-1.346)	0.991	0.998	0.77 (0.465-1.275)	0.353	0.994	1.153 (0.822-1.618)	0.419	0.680
SubApoB_LDL-3	1.01 (0.643-1.587)	0.969	0.999	0.879 (0.658-1.175)	0.384	0.956	1.271 (0.774-2.088)	0.394	0.994	1.335 (0.935-1.907)	0.120	0.448
SubApoB_LDL-4	0.957 (0.638-1.436)	0.842	0.999	0.933 (0.704-1.238)	0.632	0.998	1.401 (0.924-2.124)	0.164	0.994	1.109 (0.799-1.541)	0.546	0.731
SubApoB_LDL-5	0.884 (0.557-1.401)	0.627	0.999	1.025 (0.773-1.359)	0.866	0.998	1.081 (0.654-1.784)	0.784	0.994	1.25 (0.885-1.764)	0.216	0.564
SubApoB_LDL-6	0.827 (0.492-1.389)	0.515	0.999	0.978 (0.745-1.285)	0.874	0.998	0.948 (0.533-1.688)	0.874	0.994	1.418 (1.022-1.967)	0.044	0.331
SubTrigl_HDL-1	1.071 (0.694-1.652)	0.779	0.999	1.249 (0.925-1.687)	0.144	0.956	0.808 (0.494-1.321)	0.429	0.994	0.873 (0.612-1.246)	0.465	0.680
SubTrigl_HDL-2	1.008 (0.656-1.548)	0.974	0.999	1.327 (0.979-1.797)	0.066	0.956	0.735 (0.452-1.194)	0.245	0.994	0.861 (0.605-1.226)	0.418	0.680
SubTrigl_HDL-3	1.071 (0.711-1.615)	0.761	0.999	1.343 (0.998-1.806)	0.049	0.956	0.61 (0.372-1.002)	0.045	0.994	0.805 (0.569-1.139)	0.231	0.564
SubTrigl_HDL-4	1.057 (0.673-1.661)	0.826	0.999	1.242 (0.929-1.661)	0.142	0.956	0.609 (0.353-1.051)	0.097	0.994	0.746 (0.508-1.094)	0.144	0.511
SubChol_HDL-1	1.021 (0.636-1.64)	0.937	0.999	0.956 (0.712-1.283)	0.766	0.998	0.845 (0.504-1.418)	0.558	0.994	0.892 (0.617-1.289)	0.552	0.731
SubChol_HDL-2	1.093 (0.698-1.711)	0.721	0.999	1.004 (0.747-1.349)	0.979	0.998	0.781 (0.48-1.269)	0.354	0.994	0.725 (0.502-1.046)	0.092	0.420
SubChol_HDL-3	1.044 (0.675-1.613)	0.860	0.999	0.87 (0.644-1.176)	0.369	0.956	0.79 (0.478-1.305)	0.396	0.994	0.689 (0.472-1.004)	0.056	0.379
SubChol_HDL-4	0.9 (0.585-1.384)	0.656	0.999	0.806 (0.598-1.088)	0.159	0.956	0.942 (0.568-1.563)	0.836	0.994	0.908 (0.635-1.299)	0.610	0.790
SubFreeChol_HDL-1	0.8 (0.496-1.291)	0.401	0.999	0.933 (0.699-1.247)	0.642	0.998	1.019 (0.627-1.656)	0.943	0.994	0.976 (0.672-1.417)	0.902	0.946
SubFreeChol_HDL-2	0.769 (0.478-1.238)	0.317	0.999	0.941 (0.698-1.27)	0.694	0.998	1.248 (0.748-2.082)	0.447	0.994	0.877 (0.6-1.283)	0.512	0.714
SubFreeChol_HDL-3	0.785 (0.488-1.264)	0.351	0.999	0.892 (0.654-1.217)	0.472	0.956	0.979 (0.572-1.676)	0.945	0.994	0.886 (0.602-1.303)	0.548	0.731

SubFreeChol_HDL-4	0.836 (0.529-1.32)	0.469	0.999	0.847 (0.625-1.149)	0.285	0.956	1.138 (0.692-1.871)	0.650	0.994	1.023 (0.709-1.477)	0.904	0.946
SubPhosp_HDL-1	1.053 (0.654-1.697)	0.848	0.999	1.013 (0.754-1.361)	0.932	0.998	0.869 (0.516-1.463)	0.627	0.994	0.791 (0.539-1.16)	0.237	0.564
SubPhosp_HDL-2	1.167 (0.735-1.854)	0.552	0.999	1.043 (0.777-1.4)	0.782	0.998	0.797 (0.483-1.316)	0.412	0.994	0.633 (0.429-0.934)	0.022	0.268
SubPhosp_HDL-3	1.174 (0.755-1.825)	0.514	0.999	0.944 (0.706-1.264)	0.703	0.998	0.763 (0.454-1.28)	0.351	0.994	0.549 (0.365-0.826)	0.004	0.209
SubPhosp_HDL-4	0.964 (0.633-1.469)	0.875	0.999	0.842 (0.625-1.135)	0.259	0.956	0.967 (0.58-1.613)	0.908	0.994	0.775 (0.536-1.121)	0.188	0.564
SubApoA1_HDL-1	1.033 (0.651-1.638)	0.901	0.999	1.116 (0.829-1.503)	0.471	0.956	0.722 (0.423-1.235)	0.267	0.994	0.768 (0.523-1.129)	0.185	0.564
SubApoA1_HDL-2	1.135 (0.725-1.777)	0.618	0.999	1.016 (0.765-1.348)	0.913	0.998	0.765 (0.469-1.249)	0.319	0.994	0.644 (0.438-0.946)	0.026	0.268
SubApoA1_HDL-3	1.094 (0.707-1.692)	0.713	0.999	0.977 (0.73-1.309)	0.880	0.998	0.687 (0.41-1.152)	0.185	0.994	0.637 (0.43-0.944)	0.025	0.268
SubApoA1_HDL-4	0.862 (0.549-1.354)	0.551	0.999	0.891 (0.657-1.208)	0.458	0.956	0.784 (0.468-1.314)	0.404	0.994	0.86 (0.593-1.249)	0.443	0.680
SubApoA2_HDL-1	1.043 (0.662-1.643)	0.871	0.999	1.067 (0.807-1.41)	0.651	0.998	0.956 (0.593-1.541)	0.865	0.994	0.794 (0.55-1.145)	0.223	0.564
SubApoA2_HDL-2	1.126 (0.73-1.735)	0.631	0.999	1.052 (0.799-1.387)	0.717	0.998	0.955 (0.607-1.504)	0.855	0.994	0.829 (0.582-1.181)	0.308	0.623
SubApoA2_HDL-3	1.043 (0.667-1.632)	0.867	0.999	1.007 (0.756-1.34)	0.963	0.998	0.907 (0.556-1.48)	0.721	0.994	0.781 (0.538-1.133)	0.201	0.564
SubApoA2_HDL-4	0.83 (0.53-1.301)	0.449	0.999	0.834 (0.614-1.134)	0.246	0.956	1.047 (0.622-1.762)	0.879	0.994	0.967 (0.673-1.389)	0.860	0.925

Table S6: Effect of 24h post (t_2) rt-PA metabolites and lipids levels on early (*i.e.* sICH, non-response to thrombolysis) and late (*i.e.* three-month mortality and three-month mRS 3-6) adverse outcomes, adjusting for the major determinants for unfavourable outcomes.

	Early outcomes						Late outcomes					
	sICH			Non-response to thrombolysis			Three-month mortality			Three-month mRS 3-6		
Molecular features	OR (95% CI)	P	FDR	OR (95% CI)	P	FDR	OR (95% CI)	P	FDR	OR (95% CI)	P	FDR
Creatinine	1.46 (1.003-2.123)	0.062	0.460	0.806 (0.599-1.083)	0.147	0.469	1.346 (0.837-2.164)	0.280	0.630	1.177 (0.83-1.67)	0.379	0.745
Ala	0.806 (0.522-1.243)	0.362	0.811	1.204 (0.909-1.595)	0.196	0.469	1.211 (0.779-1.883)	0.435	0.766	1.061 (0.75-1.502)	0.745	0.818
Glu	0.844 (0.529-1.345)	0.506	0.886	0.791 (0.579-1.081)	0.142	0.469	1.289 (0.773-2.15)	0.372	0.743	1.068 (0.734-1.554)	0.736	0.818
Gln	1.009 (0.652-1.563)	0.970	0.976	1.168 (0.867-1.573)	0.309	0.469	1.09 (0.694-1.713)	0.729	0.782	0.705 (0.482-1.032)	0.074	0.445
Gly	0.956 (0.592-1.545)	0.868	0.976	0.979 (0.735-1.304)	0.886	0.886	1.183 (0.73-1.919)	0.546	0.766	0.936 (0.66-1.327)	0.717	0.818
His	1.072 (0.828-1.388)	0.640	0.886	0.9 (0.728-1.112)	0.313	0.469	1.137 (0.898-1.439)	0.274	0.630	1.091 (0.882-1.349)	0.414	0.745
Ile	0.815 (0.524-1.267)	0.406	0.811	0.914 (0.703-1.189)	0.506	0.569	0.702 (0.408-1.207)	0.257	0.630	0.949 (0.677-1.331)	0.773	0.818
Leu	1.141 (0.765-1.703)	0.559	0.886	0.854 (0.651-1.121)	0.258	0.469	1.183 (0.742-1.886)	0.553	0.766	0.916 (0.643-1.306)	0.644	0.818
Phe	1.389 (0.944-2.042)	0.128	0.460	0.722 (0.542-0.962)	0.024	0.143	1.396 (0.917-2.126)	0.147	0.604	1.243 (0.889-1.739)	0.212	0.637
Tyr	1.009 (0.691-1.473)	0.966	0.976	0.9 (0.687-1.18)	0.446	0.535	1.079 (0.716-1.626)	0.738	0.782	1.319 (0.929-1.873)	0.132	0.476
Val	0.993 (0.66-1.494)	0.976	0.976	0.894 (0.675-1.184)	0.434	0.535	1.384 (0.906-2.112)	0.168	0.604	0.94 (0.675-1.31)	0.724	0.818
Acetic acid	0.877 (0.561-1.372)	0.591	0.886	1.181 (0.883-1.579)	0.262	0.469	0.851 (0.559-1.294)	0.472	0.766	1.651 (1.133-2.407)	0.010	0.173
Citric acid	1.019 (0.688-1.508)	0.931	0.976	0.911 (0.669-1.241)	0.555	0.587	1.408 (0.956-2.073)	0.115	0.604	1.237 (0.858-1.783)	0.265	0.682
Lactic acid	1.245 (0.83-1.869)	0.352	0.811	0.842 (0.642-1.103)	0.213	0.469	1.468 (1.001-2.153)	0.065	0.604	1.302 (0.934-1.814)	0.121	0.476
3-HB	1.382 (0.953-2.004)	0.115	0.460	0.571 (0.413-0.791)	0.000	0.007	1.108 (0.756-1.625)	0.634	0.782	1.434 (1.013-2.032)	0.045	0.403
Acetone	1.223 (0.886-1.688)	0.246	0.738	0.609 (0.43-0.863)	0.003	0.026	1.042 (0.757-1.433)	0.813	0.813	1.177 (0.831-1.666)	0.377	0.745
Pyruvic acid	1.453 (0.962-2.194)	0.105	0.460	0.83 (0.633-1.088)	0.177	0.469	1.426 (0.927-2.196)	0.145	0.604	0.882 (0.626-1.243)	0.486	0.796
Glucose	1.589 (1.002-2.519)	0.071	0.460	0.846 (0.582-1.23)	0.385	0.533	0.867 (0.477-1.574)	0.674	0.782	1.051 (0.677-1.633)	0.829	0.829
Trigl	0.483 (0.241-0.968)	0.044	0.276	1.16 (0.858-1.568)	0.334	0.686	1.3 (0.708-2.385)	0.483	0.987	1.363 (0.924-2.012)	0.133	0.353
Chol	0.813 (0.511-1.294)	0.421	0.819	0.899 (0.672-1.204)	0.477	0.724	1.151 (0.67-1.978)	0.651	0.987	1.376 (0.939-2.018)	0.111	0.327
LDL-Chol	1.025 (0.655-1.605)	0.920	0.973	0.841 (0.629-1.125)	0.243	0.686	1.092 (0.653-1.825)	0.766	0.987	1.461 (0.997-2.14)	0.056	0.280
HDL-Chol	1.075 (0.707-1.636)	0.754	0.961	0.861 (0.644-1.151)	0.313	0.686	1.018 (0.61-1.701)	0.950	0.987	0.837 (0.584-1.199)	0.344	0.560
Apo-A1	0.861 (0.549-1.35)	0.547	0.890	1.035 (0.762-1.405)	0.829	0.976	0.713 (0.424-1.2)	0.248	0.987	0.765 (0.519-1.127)	0.186	0.436
Apo-A2	0.879 (0.555-1.393)	0.616	0.936	0.99 (0.728-1.344)	0.946	0.976	0.927 (0.554-1.551)	0.795	0.987	0.993 (0.68-1.452)	0.973	0.990
Apo-B100	0.897 (0.572-1.407)	0.663	0.946	0.889 (0.67-1.179)	0.415	0.696	1.143 (0.689-1.896)	0.643	0.987	1.595 (1.087-2.339)	0.018	0.175
Apo-B100-Apo-A1	1.054 (0.714-1.556)	0.807	0.973	0.975 (0.74-1.285)	0.857	0.976	1.261 (0.818-1.945)	0.332	0.987	1.738 (1.17-2.583)	0.006	0.076
VLDL_PN	0.636 (0.371-1.092)	0.114	0.511	0.901 (0.682-1.19)	0.462	0.714	1.236 (0.711-2.147)	0.512	0.987	1.198 (0.813-1.766)	0.378	0.580

IDL_PN	0.829 (0.531-1.295)	0.438	0.819	0.889 (0.67-1.179)	0.415	0.696	1.095 (0.684-1.752)	0.731	0.987	1.403 (0.966-2.037)	0.084	0.280
LDL_PN	1.001 (0.646-1.552)	0.997	0.997	1.235 (0.92-1.659)	0.159	0.686	1.13 (0.688-1.858)	0.664	0.987	1.524 (1.044-2.226)	0.031	0.246
LDL1_PN	1.178 (0.77-1.801)	0.489	0.870	1.013 (0.769-1.335)	0.926	0.976	1.005 (0.625-1.616)	0.985	0.987	1.11 (0.765-1.61)	0.593	0.727
LDL2_PN	1.426 (0.883-2.303)	0.176	0.558	0.846 (0.637-1.123)	0.246	0.686	0.751 (0.428-1.317)	0.374	0.987	1.164 (0.799-1.696)	0.441	0.606
LDL3_PN	1.515 (0.937-2.449)	0.123	0.511	0.858 (0.635-1.16)	0.320	0.686	0.971 (0.562-1.679)	0.928	0.987	1.448 (0.993-2.112)	0.061	0.280
LDL4_PN	0.98 (0.647-1.486)	0.930	0.973	0.857 (0.631-1.163)	0.322	0.686	1.189 (0.681-2.075)	0.597	0.987	1.17 (0.817-1.674)	0.410	0.592
LDL5_PN	0.786 (0.491-1.257)	0.347	0.791	0.78 (0.582-1.044)	0.094	0.686	1.035 (0.601-1.783)	0.912	0.987	1.289 (0.882-1.884)	0.203	0.436
LDL6_PN	0.658 (0.379-1.142)	0.161	0.540	0.962 (0.723-1.281)	0.791	0.976	1.797 (1.056-3.055)	0.069	0.982	1.79 (1.233-2.597)	0.002	0.040
LMF_Trigl_VLDL	0.405 (0.19-0.867)	0.020	0.178	0.994 (0.741-1.333)	0.968	0.976	1.604 (0.873-2.948)	0.205	0.987	1.488 (1.004-2.206)	0.055	0.280
LMF_Trigl_IDL	0.487 (0.247-0.962)	0.040	0.271	0.899 (0.676-1.196)	0.463	0.714	1.123 (0.588-2.145)	0.773	0.987	1.198 (0.809-1.776)	0.383	0.580
LMF_Trigl_LDL	1.024 (0.713-1.471)	0.905	0.973	1.163 (0.855-1.582)	0.336	0.686	0.983 (0.668-1.447)	0.935	0.987	1.278 (0.884-1.848)	0.195	0.436
LMF_Trigl_HDL	0.743 (0.477-1.156)	0.223	0.604	1.194 (0.878-1.623)	0.257	0.686	0.526 (0.299-0.927)	0.048	0.982	0.797 (0.553-1.147)	0.235	0.490
LMF_Chol_VLDL	0.452 (0.251-0.812)	0.010	0.128	0.942 (0.721-1.231)	0.665	0.960	1.093 (0.61-1.959)	0.792	0.987	1.161 (0.798-1.688)	0.450	0.611
LMF_Chol_IDL	0.711 (0.441-1.146)	0.187	0.563	1.316 (0.971-1.785)	0.074	0.686	1.085 (0.655-1.796)	0.775	0.987	1.5 (1.034-2.177)	0.037	0.246
LMF_Chol_LDL	1.025 (0.655-1.605)	0.920	0.973	1.247 (0.929-1.674)	0.139	0.686	1.092 (0.653-1.825)	0.766	0.987	1.461 (0.997-2.14)	0.056	0.280
LMF_Chol_HDL	1.075 (0.707-1.636)	0.754	0.961	1.017 (0.768-1.345)	0.909	0.976	1.018 (0.61-1.701)	0.950	0.987	0.837 (0.584-1.199)	0.344	0.560
LMF_FreeChol_VLDL	0.419 (0.222-0.793)	0.009	0.128	0.841 (0.629-1.125)	0.243	0.686	1.394 (0.755-2.575)	0.365	0.987	1.244 (0.844-1.834)	0.286	0.515
LMF_FreeChol_IDL	0.725 (0.45-1.167)	0.212	0.604	0.861 (0.644-1.151)	0.313	0.686	1.014 (0.606-1.695)	0.963	0.987	1.414 (0.974-2.054)	0.076	0.280
LMF_FreeChol_LDL	1.039 (0.657-1.644)	0.882	0.973	1.23 (0.908-1.667)	0.179	0.686	1.159 (0.678-1.98)	0.636	0.987	1.532 (1.043-2.251)	0.033	0.246
LMF_FreeChol_HDL	0.904 (0.59-1.385)	0.670	0.946	1.044 (0.788-1.384)	0.763	0.976	1.18 (0.705-1.975)	0.586	0.987	1.045 (0.729-1.498)	0.818	0.897
LMF_Phosp_VLDL	0.382 (0.194-0.753)	0.006	0.128	0.801 (0.597-1.073)	0.136	0.686	1.575 (0.845-2.934)	0.227	0.987	1.207 (0.809-1.8)	0.373	0.580
LMF_Phosp_IDL	0.647 (0.384-1.09)	0.120	0.511	0.853 (0.641-1.134)	0.272	0.686	1.065 (0.607-1.869)	0.846	0.987	1.336 (0.915-1.951)	0.147	0.372
LMF_Phosp_LDL	1.067 (0.683-1.669)	0.795	0.973	1.293 (0.946-1.767)	0.104	0.686	1.06 (0.635-1.769)	0.843	0.987	1.369 (0.937-1.998)	0.112	0.327
LMF_Phosp_HDL	1.111 (0.708-1.744)	0.673	0.946	1.058 (0.793-1.411)	0.705	0.976	0.747 (0.436-1.279)	0.337	0.987	0.669 (0.458-0.977)	0.041	0.257
LMF_ApoA1_HDL	0.894 (0.575-1.39)	0.644	0.946	0.836 (0.625-1.119)	0.228	0.686	0.675 (0.396-1.152)	0.189	0.987	0.718 (0.489-1.055)	0.098	0.311
LMF_ApoA2_HDL	0.902 (0.566-1.436)	0.691	0.949	0.898 (0.662-1.219)	0.493	0.740	0.952 (0.561-1.616)	0.871	0.987	1 (0.683-1.467)	0.998	0.998
LMF_ApoB_VLDL	0.636 (0.371-1.092)	0.114	0.511	1.021 (0.752-1.385)	0.894	0.976	1.236 (0.712-2.148)	0.511	0.987	1.198 (0.813-1.766)	0.378	0.580
LMF_ApoB_IDL	0.829 (0.53-1.295)	0.438	0.819	0.985 (0.725-1.337)	0.921	0.976	1.095 (0.684-1.752)	0.731	0.987	1.403 (0.966-2.038)	0.083	0.280
LMF_ApoB_LDL	1.001 (0.646-1.552)	0.997	0.997	1.236 (0.92-1.659)	0.159	0.686	1.13 (0.688-1.858)	0.664	0.987	1.524 (1.044-2.226)	0.031	0.246
SubTrigl_VLDL-1	0.458 (0.198-1.057)	0.075	0.426	1.013 (0.769-1.335)	0.926	0.976	1.685 (0.905-3.138)	0.183	0.987	1.533 (1.044-2.251)	0.037	0.246
SubTrigl_VLDL-2	0.372 (0.193-0.717)	0.003	0.097	0.846 (0.637-1.123)	0.246	0.686	1.574 (0.866-2.861)	0.205	0.987	1.275 (0.861-1.888)	0.242	0.490
SubTrigl_VLDL-3	0.44 (0.242-0.803)	0.008	0.128	1.085 (0.797-1.476)	0.604	0.883	1.476 (0.817-2.667)	0.263	0.987	1.315 (0.886-1.953)	0.188	0.436
SubTrigl_VLDL-4	0.656 (0.393-1.096)	0.126	0.511	1.233 (0.908-1.675)	0.179	0.686	1.415 (0.808-2.477)	0.287	0.987	1.239 (0.843-1.821)	0.289	0.515
SubTrigl_VLDL-5	0.917 (0.594-1.416)	0.720	0.961	1.271 (0.939-1.72)	0.119	0.686	1.341 (0.776-2.319)	0.361	0.987	0.833 (0.584-1.189)	0.331	0.559
SubChol_VLDL-1	0.395 (0.179-0.869)	0.024	0.194	1.213 (0.906-1.622)	0.194	0.686	1.244 (0.655-2.365)	0.583	0.987	1.364 (0.932-1.996)	0.128	0.353
SubChol_VLDL-2	0.383 (0.203-0.719)	0.003	0.097	1.121 (0.845-1.488)	0.429	0.696	1.044 (0.577-1.889)	0.900	0.987	1.106 (0.764-1.6)	0.607	0.729
SubChol_VLDL-3	0.465 (0.262-0.825)	0.010	0.128	1.137 (0.839-1.54)	0.408	0.696	1.151 (0.657-2.016)	0.662	0.987	1.341 (0.925-1.945)	0.133	0.353
SubChol_VLDL-4	0.575 (0.349-0.947)	0.038	0.270	1.252 (0.936-1.674)	0.127	0.686	1.014 (0.604-1.7)	0.963	0.987	1.075 (0.75-1.542)	0.704	0.810
SubChol_VLDL-5	1.005 (0.649-1.556)	0.984	0.997	1.188 (0.89-1.586)	0.242	0.686	0.771 (0.444-1.342)	0.417	0.987	0.497 (0.334-0.738)	0.000	0.040
SubFreeChol_VLDL-1	0.424 (0.195-0.923)	0.031	0.237	1.214 (0.915-1.612)	0.178	0.686	1.131 (0.584-2.191)	0.763	0.987	1.305 (0.886-1.92)	0.198	0.436
SubFreeChol_VLDL-2	0.46 (0.249-0.852)	0.014	0.164	1.226 (0.92-1.633)	0.163	0.686	1.283 (0.718-2.293)	0.471	0.987	1.326 (0.906-1.942)	0.159	0.394
SubFreeChol_VLDL-3	0.468 (0.253-0.868)	0.018	0.171	1.173 (0.869-1.584)	0.296	0.686	1.31 (0.735-2.336)	0.432	0.987	1.441 (0.984-2.11)	0.068	0.280
SubFreeChol_VLDL-4	0.681 (0.425-1.09)	0.130	0.511	1.239 (0.922-1.665)	0.153	0.686	1.071 (0.648-1.77)	0.808	0.987	1.202 (0.837-1.726)	0.334	0.559
SubFreeChol_VLDL-5	0.732 (0.411-1.304)	0.336	0.791	1.183 (0.882-1.586)	0.262	0.686	1.362 (0.743-2.496)	0.393	0.987	0.975 (0.645-1.473)	0.907	0.949
SubPhosp_VLDL-1	0.361 (0.157-0.832)	0.017	0.171	1.145 (0.864-1.517)	0.346	0.686	1.389 (0.735-2.624)	0.405	0.987	1.457 (0.984-2.157)	0.071	0.280
SubPhosp_VLDL-2	0.364 (0.193-0.687)	0.001	0.097	1.157 (0.849-1.577)	0.354	0.686	1.346 (0.74-2.446)	0.406	0.987	1.251 (0.853-1.834)	0.268	0.501
SubPhosp_VLDL-3	0.46 (0.257-0.822)	0.010	0.128	1.14 (0.838-1.551)	0.406	0.696	1.292 (0.729-2.289)	0.447	0.987	1.31 (0.891-1.925)	0.181	0.436

SubPhosp_VLDL-4	0.646 (0.396-1.052)	0.096	0.511	1.275 (0.942-1.726)	0.113	0.686	1.132 (0.667-1.922)	0.682	0.987	1.088 (0.752-1.575)	0.664	0.780
SubPhosp_VLDL-5	0.812 (0.525-1.255)	0.389	0.819	1.292 (0.96-1.739)	0.089	0.686	0.841 (0.493-1.435)	0.577	0.987	0.585 (0.401-0.852)	0.006	0.076
SubTrigl_LDL-1	1.082 (0.765-1.53)	0.680	0.946	1.224 (0.921-1.627)	0.164	0.686	0.97 (0.652-1.443)	0.887	0.987	1.131 (0.787-1.625)	0.518	0.667
SubTrigl_LDL-2	1.155 (0.825-1.617)	0.437	0.819	1.323 (0.992-1.766)	0.055	0.686	1.026 (0.711-1.48)	0.897	0.987	1.099 (0.772-1.564)	0.614	0.729
SubTrigl_LDL-3	1.166 (0.818-1.661)	0.437	0.819	0.975 (0.742-1.281)	0.855	0.976	1.063 (0.727-1.553)	0.770	0.987	1.057 (0.755-1.479)	0.754	0.857
SubTrigl_LDL-4	1.01 (0.695-1.469)	0.960	0.995	0.953 (0.723-1.257)	0.734	0.976	1.016 (0.681-1.517)	0.941	0.987	1.055 (0.744-1.495)	0.773	0.864
SubTrigl_LDL-5	0.803 (0.515-1.25)	0.358	0.801	0.883 (0.669-1.166)	0.379	0.696	1.041 (0.665-1.629)	0.873	0.987	1.159 (0.819-1.642)	0.420	0.598
SubTrigl_LDL-6	0.706 (0.405-1.231)	0.249	0.661	0.992 (0.756-1.302)	0.954	0.976	1.751 (1.182-2.594)	0.010	0.982	1.745 (1.213-2.508)	0.001	0.040
SubChol_LDL-1	1.162 (0.728-1.856)	0.565	0.895	1.024 (0.782-1.342)	0.864	0.976	0.936 (0.551-1.592)	0.827	0.987	1.043 (0.715-1.52)	0.834	0.905
SubChol_LDL-2	1.405 (0.874-2.259)	0.192	0.563	0.782 (0.582-1.051)	0.092	0.686	0.802 (0.457-1.406)	0.498	0.987	1.169 (0.806-1.695)	0.425	0.598
SubChol_LDL-3	1.451 (0.909-2.317)	0.154	0.540	0.879 (0.648-1.194)	0.413	0.696	1.036 (0.601-1.786)	0.911	0.987	1.406 (0.968-2.04)	0.081	0.280
SubChol_LDL-4	1.004 (0.665-1.514)	0.987	0.997	0.845 (0.624-1.144)	0.277	0.686	1.188 (0.682-2.069)	0.596	0.987	1.146 (0.799-1.645)	0.475	0.630
SubChol_LDL-5	0.811 (0.504-1.306)	0.420	0.819	0.803 (0.602-1.07)	0.133	0.686	1.071 (0.613-1.872)	0.830	0.987	1.258 (0.854-1.851)	0.260	0.493
SubChol_LDL-6	0.691 (0.41-1.165)	0.190	0.563	0.959 (0.718-1.28)	0.775	0.976	1.804 (1.084-3.003)	0.042	0.982	1.743 (1.207-2.516)	0.003	0.049
SubFreeChol_LDL-1	1.082 (0.682-1.718)	0.759	0.961	1.006 (0.746-1.358)	0.967	0.976	0.92 (0.546-1.552)	0.777	0.987	1.127 (0.773-1.643)	0.546	0.691
SubFreeChol_LDL-2	1.213 (0.778-1.894)	0.428	0.819	0.888 (0.667-1.184)	0.418	0.696	0.873 (0.508-1.499)	0.662	0.987	1.253 (0.864-1.818)	0.246	0.490
SubFreeChol_LDL-3	1.538 (0.939-2.52)	0.120	0.511	0.874 (0.644-1.185)	0.388	0.696	1.077 (0.622-1.865)	0.817	0.987	1.427 (0.975-2.086)	0.073	0.280
SubFreeChol_LDL-4	1.114 (0.723-1.717)	0.652	0.946	0.852 (0.63-1.153)	0.301	0.686	1.099 (0.633-1.909)	0.769	0.987	1.337 (0.925-1.931)	0.134	0.353
SubFreeChol_LDL-5	0.879 (0.553-1.399)	0.615	0.936	0.755 (0.562-1.014)	0.060	0.686	1.075 (0.628-1.839)	0.814	0.987	1.444 (0.978-2.13)	0.071	0.280
SubFreeChol_LDL-6	0.767 (0.487-1.207)	0.281	0.728	0.855 (0.639-1.144)	0.291	0.686	1.267 (0.771-2.081)	0.400	0.987	1.844 (1.266-2.687)	0.001	0.040
SubPhosp_LDL-1	1.181 (0.756-1.843)	0.504	0.870	0.906 (0.673-1.221)	0.519	0.768	0.935 (0.562-1.557)	0.814	0.987	1.007 (0.693-1.465)	0.971	0.990
SubPhosp_LDL-2	1.483 (0.903-2.434)	0.147	0.540	0.84 (0.631-1.118)	0.230	0.686	0.781 (0.439-1.389)	0.458	0.987	1.121 (0.768-1.636)	0.567	0.710
SubPhosp_LDL-3	1.492 (0.919-2.422)	0.140	0.533	0.885 (0.652-1.2)	0.434	0.696	0.962 (0.553-1.672)	0.904	0.987	1.342 (0.921-1.954)	0.136	0.353
SubPhosp_LDL-4	1.025 (0.682-1.541)	0.912	0.973	0.841 (0.618-1.145)	0.272	0.686	1.171 (0.675-2.033)	0.624	0.987	1.138 (0.792-1.635)	0.501	0.657
SubPhosp_LDL-5	0.817 (0.511-1.306)	0.430	0.819	0.796 (0.595-1.065)	0.123	0.686	1.009 (0.579-1.759)	0.978	0.987	1.19 (0.813-1.742)	0.387	0.580
SubPhosp_LDL-6	0.712 (0.429-1.184)	0.218	0.604	0.959 (0.718-1.281)	0.778	0.976	1.764 (1.056-2.947)	0.063	0.982	1.701 (1.184-2.445)	0.004	0.061
SubApoB_LDL-1	1.178 (0.77-1.801)	0.489	0.870	1.015 (0.754-1.366)	0.921	0.976	1.005 (0.625-1.616)	0.985	0.987	1.11 (0.765-1.61)	0.593	0.727
SubApoB_LDL-2	1.425 (0.882-2.302)	0.176	0.558	0.875 (0.657-1.164)	0.356	0.686	0.751 (0.428-1.316)	0.374	0.987	1.164 (0.799-1.695)	0.441	0.606
SubApoB_LDL-3	1.515 (0.937-2.449)	0.123	0.511	0.858 (0.635-1.16)	0.320	0.686	0.972 (0.562-1.68)	0.928	0.987	1.448 (0.992-2.112)	0.061	0.280
SubApoB_LDL-4	0.98 (0.647-1.486)	0.930	0.973	0.857 (0.632-1.163)	0.323	0.686	1.189 (0.681-2.076)	0.597	0.987	1.17 (0.817-1.674)	0.410	0.592
SubApoB_LDL-5	0.786 (0.491-1.258)	0.347	0.791	0.78 (0.583-1.044)	0.094	0.686	1.036 (0.601-1.784)	0.911	0.987	1.289 (0.882-1.884)	0.203	0.436
SubApoB_LDL-6	0.658 (0.379-1.142)	0.161	0.540	0.962 (0.722-1.281)	0.791	0.976	1.797 (1.057-3.056)	0.069	0.982	1.79 (1.233-2.597)	0.002	0.040
SubTrigl_HDL-1	0.839 (0.526-1.338)	0.497	0.870	0.994 (0.741-1.333)	0.967	0.976	0.692 (0.39-1.227)	0.255	0.987	0.948 (0.649-1.383)	0.787	0.871
SubTrigl_HDL-2	0.83 (0.542-1.271)	0.426	0.819	0.899 (0.676-1.196)	0.463	0.714	0.601 (0.347-1.041)	0.099	0.987	0.909 (0.635-1.302)	0.613	0.729
SubTrigl_HDL-3	0.784 (0.512-1.202)	0.299	0.757	1.161 (0.85-1.586)	0.348	0.686	0.543 (0.309-0.952)	0.052	0.982	0.868 (0.606-1.244)	0.456	0.611
SubTrigl_HDL-4	0.619 (0.385-0.994)	0.060	0.363	1.193 (0.884-1.61)	0.249	0.686	0.527 (0.296-0.936)	0.057	0.982	0.681 (0.455-1.018)	0.066	0.280
SubChol_HDL-1	1.022 (0.655-1.594)	0.930	0.973	1.325 (0.981-1.79)	0.064	0.686	1.213 (0.704-2.089)	0.535	0.987	1.08 (0.743-1.569)	0.696	0.810
SubChol_HDL-2	1.276 (0.826-1.974)	0.307	0.761	1.559 (1.135-2.143)	0.005	0.586	0.974 (0.575-1.65)	0.929	0.987	0.806 (0.561-1.16)	0.257	0.493
SubChol_HDL-3	1.167 (0.761-1.788)	0.516	0.878	0.853 (0.633-1.149)	0.297	0.686	0.917 (0.558-1.506)	0.759	0.987	0.889 (0.628-1.26)	0.520	0.667
SubChol_HDL-4	0.972 (0.622-1.52)	0.910	0.973	0.814 (0.6-1.105)	0.186	0.686	1.036 (0.608-1.765)	0.907	0.987	0.836 (0.567-1.233)	0.379	0.580
SubFreeChol_HDL-1	0.962 (0.62-1.494)	0.875	0.973	0.865 (0.646-1.159)	0.334	0.686	1.148 (0.686-1.923)	0.640	0.987	1.256 (0.873-1.808)	0.238	0.490
SubFreeChol_HDL-2	1.092 (0.69-1.728)	0.735	0.961	0.978 (0.719-1.33)	0.886	0.976	1.101 (0.631-1.919)	0.768	0.987	1.247 (0.866-1.794)	0.249	0.490
SubFreeChol_HDL-3	0.852 (0.535-1.358)	0.539	0.890	0.817 (0.612-1.092)	0.171	0.686	0.811 (0.473-1.388)	0.494	0.987	1.014 (0.693-1.483)	0.946	0.980
SubFreeChol_HDL-4	0.921 (0.582-1.457)	0.745	0.961	0.741 (0.546-1.005)	0.051	0.686	0.985 (0.563-1.722)	0.962	0.987	1.027 (0.701-1.505)	0.894	0.944
SubPhosp_HDL-1	1.045 (0.656-1.663)	0.865	0.973	0.942 (0.697-1.273)	0.699	0.976	0.992 (0.565-1.741)	0.981	0.987	0.94 (0.638-1.383)	0.760	0.857
SubPhosp_HDL-2	1.283 (0.815-2.02)	0.318	0.771	0.947 (0.699-1.282)	0.723	0.976	0.804 (0.462-1.4)	0.487	0.987	0.718 (0.492-1.046)	0.090	0.294
SubPhosp_HDL-3	1.153 (0.741-1.795)	0.561	0.895	0.883 (0.65-1.2)	0.429	0.696	0.689 (0.412-1.153)	0.203	0.987	0.701 (0.486-1.011)	0.062	0.280

SubPhosp_HDL-4	0.954 (0.613-1.483)	0.845	0.973	0.864 (0.632-1.181)	0.361	0.686	0.831 (0.484-1.427)	0.547	0.987	0.693 (0.465-1.033)	0.076	0.280
SubApoA1_HDL-1	0.959 (0.605-1.52)	0.869	0.973	0.949 (0.705-1.277)	0.729	0.976	0.731 (0.413-1.292)	0.327	0.987	0.808 (0.544-1.199)	0.301	0.527
SubApoA1_HDL-2	1.041 (0.665-1.628)	0.872	0.973	1.044 (0.768-1.419)	0.786	0.976	0.747 (0.45-1.241)	0.311	0.987	0.726 (0.499-1.055)	0.101	0.311
SubApoA1_HDL-3	1.033 (0.66-1.618)	0.895	0.973	0.977 (0.721-1.323)	0.881	0.976	0.817 (0.475-1.404)	0.515	0.987	0.813 (0.564-1.17)	0.278	0.511
SubApoA1_HDL-4	0.825 (0.52-1.308)	0.448	0.824	1.003 (0.746-1.349)	0.984	0.984	0.887 (0.515-1.528)	0.699	0.987	0.808 (0.539-1.21)	0.313	0.541
SubApoA2_HDL-1	0.928 (0.577-1.493)	0.779	0.973	0.967 (0.717-1.303)	0.826	0.976	0.904 (0.513-1.591)	0.755	0.987	0.999 (0.685-1.456)	0.994	0.998
SubApoA2_HDL-2	0.964 (0.599-1.55)	0.891	0.973	1.063 (0.775-1.457)	0.707	0.976	1.005 (0.57-1.774)	0.987	0.987	1.176 (0.815-1.698)	0.404	0.592
SubApoA2_HDL-3	0.909 (0.566-1.459)	0.720	0.961	0.979 (0.729-1.315)	0.889	0.976	0.818 (0.48-1.392)	0.509	0.987	1.033 (0.712-1.498)	0.870	0.926
SubApoA2_HDL-4	0.869 (0.549-1.376)	0.581	0.907	0.988 (0.737-1.325)	0.937	0.976	1.047 (0.612-1.793)	0.881	0.987	0.963 (0.651-1.424)	0.853	0.917

Table S7: Effect of pre-post-rtPA variations of metabolites and lipids levels on early (*i.e.* sICH, non-response to thrombolysis) and late (*i.e.* three-month mortality and three-month mRS 3-6) adverse outcomes, adjusting for the major determinants for unfavourable outcomes.

	**Early outcomes**						**Late outcomes**					
	**sICH**			**Non-response to thrombolysis**			**Three-month mortality**			**Three-month mRS 3-6**		
**Molecular features**	**OR (95% CI)**	**P**	**FDR**	**OR (95% CI)**	**P**	**FDR**	**OR (95% CI)**	**P**	**FDR**	**OR (95% CI)**	**P**	**FDR**
Creatinine	1.285 (0.887-1.861)	0.229	0.796	0.755 (0.568-1.003)	0.047	0.238	0.691 (0.429-1.114)	0.166	0.819	1.185 (0.837-1.677)	0.365	0.766
Ala	0.919 (0.603-1.401)	0.719	0.846	1.327 (0.994-1.771)	0.053	0.238	1.031 (0.667-1.595)	0.898	0.957	0.909 (0.645-1.282)	0.596	0.766
Glu	0.925 (0.593-1.442)	0.752	0.846	0.822 (0.605-1.116)	0.208	0.558	1.042 (0.654-1.66)	0.876	0.957	1.565 (1.069-2.29)	0.023	0.412
Gln	0.889 (0.596-1.325)	0.619	0.796	1.285 (0.922-1.791)	0.132	0.474	1.083 (0.763-1.535)	0.680	0.957	0.864 (0.612-1.221)	0.413	0.766
Gly	1.245 (0.814-1.904)	0.351	0.796	1.04 (0.789-1.371)	0.783	0.888	0.786 (0.489-1.263)	0.362	0.930	0.903 (0.639-1.276)	0.576	0.766
His	0.996 (0.666-1.488)	0.984	0.984	0.938 (0.711-1.237)	0.652	0.888	1.165 (0.784-1.731)	0.481	0.957	1.127 (0.804-1.579)	0.508	0.766
Ile	0.803 (0.549-1.174)	0.292	0.796	0.986 (0.756-1.287)	0.919	0.919	0.678 (0.448-1.024)	0.084	0.819	0.911 (0.657-1.262)	0.585	0.766
Leu	1.176 (0.791-1.749)	0.457	0.796	0.946 (0.727-1.232)	0.683	0.888	0.832 (0.55-1.257)	0.422	0.949	0.833 (0.596-1.165)	0.302	0.766
Phe	1.736 (1.157-2.605)	0.015	0.274	0.86 (0.654-1.131)	0.280	0.558	1.133 (0.735-1.746)	0.610	0.957	1.023 (0.73-1.433)	0.898	0.898
Tyr	1.125 (0.759-1.668)	0.596	0.796	0.972 (0.742-1.274)	0.839	0.888	0.937 (0.63-1.392)	0.764	0.957	0.948 (0.69-1.301)	0.746	0.790
Val	1.119 (0.749-1.671)	0.614	0.796	0.92 (0.703-1.204)	0.543	0.888	0.969 (0.637-1.474)	0.893	0.957	0.894 (0.649-1.231)	0.503	0.766
Acetic acid	0.993 (0.611-1.615)	0.980	0.984	0.962 (0.719-1.286)	0.793	0.888	0.965 (0.575-1.62)	0.904	0.957	1.45 (0.972-2.165)	0.073	0.527
Citric acid	1.239 (0.808-1.901)	0.362	0.796	0.832 (0.614-1.13)	0.239	0.558	1.265 (0.829-1.931)	0.302	0.907	1.399 (0.958-2.044)	0.088	0.527
Lactic acid	1.55 (0.969-2.479)	0.093	0.417	0.853 (0.642-1.134)	0.275	0.558	1.461 (0.886-2.41)	0.182	0.819	1.272 (0.895-1.808)	0.192	0.766
3-HB	1.214 (0.783-1.883)	0.423	0.796	0.725 (0.543-0.969)	0.028	0.238	1.394 (0.823-2.361)	0.259	0.907	1.238 (0.867-1.768)	0.250	0.766
Acetone	1.162 (0.753-1.794)	0.531	0.796	0.684 (0.516-0.906)	0.007	0.129	1.073 (0.668-1.724)	0.790	0.957	1.226 (0.865-1.738)	0.261	0.766
Pyruvic acid	1.692 (1.062-2.695)	0.035	0.297	0.86 (0.643-1.15)	0.310	0.558	1.512 (0.943-2.425)	0.107	0.819	0.927 (0.646-1.33)	0.689	0.775
Glucose	1.68 (1.042-2.707)	0.050	0.297	0.955 (0.717-1.272)	0.757	0.888	1.006 (0.618-1.636)	0.984	0.984	0.926 (0.671-1.279)	0.649	0.775
Trigl	0.651 (0.431-0.985)	0.057	0.339	0.961 (0.72-1.283)	0.788	0.990	1.179 (0.741-1.877)	0.513	0.976	1.148 (0.791-1.665)	0.479	0.973
Chol	0.91 (0.599-1.382)	0.681	0.955	0.948 (0.718-1.253)	0.711	0.990	1.15 (0.761-1.737)	0.543	0.976	1.172 (0.827-1.662)	0.387	0.973
LDL-Chol	0.871 (0.586-1.295)	0.528	0.955	0.976 (0.735-1.297)	0.868	0.990	0.964 (0.648-1.433)	0.865	0.976	0.906 (0.63-1.305)	0.610	0.973

HDL-Chol	1.121 (0.738-1.703)	0.621	0.955	0.938 (0.708-1.244)	0.659	0.990	1.242 (0.776-1.99)	0.411	0.976	1.209 (0.841-1.739)	0.320	0.973
Apo-A1	0.869 (0.575-1.313)	0.538	0.955	0.997 (0.756-1.315)	0.981	0.990	1.002 (0.659-1.525)	0.992	0.992	1.221 (0.85-1.755)	0.295	0.973
Apo-A2	1.044 (0.695-1.568)	0.851	0.980	1.037 (0.79-1.362)	0.793	0.990	0.926 (0.613-1.397)	0.734	0.976	1.244 (0.882-1.756)	0.225	0.973
Apo-B100	0.909 (0.606-1.363)	0.669	0.955	0.785 (0.592-1.041)	0.090	0.990	1.08 (0.715-1.632)	0.734	0.976	1.104 (0.778-1.565)	0.593	0.973
Apo-B100-Apo-A1	1.015 (0.656-1.571)	0.952	0.997	1.016 (0.767-1.346)	0.914	0.990	1.088 (0.697-1.7)	0.737	0.976	0.933 (0.652-1.335)	0.720	0.979
VLDL_PN	0.597 (0.39-0.914)	0.024	0.301	0.776 (0.58-1.038)	0.081	0.990	1.095 (0.687-1.747)	0.723	0.976	1.044 (0.724-1.505)	0.822	0.979
IDL_PN	0.87 (0.591-1.283)	0.517	0.955	0.785 (0.592-1.041)	0.090	0.990	0.917 (0.603-1.395)	0.710	0.976	1.268 (0.904-1.779)	0.177	0.973
LDL_PN	1.054 (0.709-1.567)	0.811	0.955	0.943 (0.704-1.265)	0.698	0.990	0.918 (0.601-1.402)	0.714	0.976	1.025 (0.709-1.483)	0.898	0.985
LDL1_PN	1.092 (0.728-1.638)	0.698	0.955	0.842 (0.642-1.106)	0.216	0.990	0.864 (0.525-1.421)	0.612	0.976	1.161 (0.819-1.646)	0.420	0.973
LDL2_PN	1.023 (0.687-1.525)	0.917	0.987	0.839 (0.632-1.114)	0.225	0.990	0.764 (0.468-1.249)	0.334	0.976	0.956 (0.68-1.343)	0.800	0.979
LDL3_PN	1.237 (0.8-1.913)	0.388	0.955	0.875 (0.668-1.146)	0.330	0.990	0.725 (0.458-1.149)	0.193	0.976	1.034 (0.712-1.503)	0.866	0.979
LDL4_PN	0.769 (0.506-1.171)	0.257	0.826	0.873 (0.646-1.18)	0.376	0.990	1.192 (0.744-1.91)	0.506	0.976	1.093 (0.764-1.563)	0.637	0.973
LDL5_PN	0.827 (0.564-1.214)	0.370	0.955	0.765 (0.567-1.032)	0.076	0.990	0.752 (0.51-1.108)	0.170	0.976	0.881 (0.637-1.219)	0.454	0.973
LDL6_PN	0.97 (0.659-1.428)	0.887	0.982	1.01 (0.766-1.331)	0.944	0.990	1.31 (0.83-2.069)	0.275	0.976	1.197 (0.846-1.694)	0.332	0.973
LMF_Trigl_VLDL	0.639 (0.418-0.977)	0.051	0.339	0.969 (0.733-1.28)	0.824	0.990	1.21 (0.764-1.918)	0.443	0.976	1.188 (0.821-1.721)	0.373	0.973
LMF_Trigl_IDL	0.609 (0.383-0.97)	0.047	0.339	0.87 (0.652-1.162)	0.346	0.990	0.951 (0.615-1.471)	0.834	0.976	1.267 (0.887-1.808)	0.203	0.973
LMF_Trigl_LDL	0.944 (0.656-1.358)	0.775	0.955	0.976 (0.729-1.306)	0.870	0.990	0.609 (0.414-0.897)	0.023	0.934	1.035 (0.734-1.458)	0.854	0.979
LMF_Trigl_HDL	0.803 (0.574-1.124)	0.245	0.826	0.972 (0.727-1.3)	0.851	0.990	0.597 (0.404-0.882)	0.031	0.934	0.885 (0.664-1.18)	0.417	0.973
LMF_Chol_VLDL	0.611 (0.405-0.921)	0.026	0.301	0.808 (0.611-1.067)	0.123	0.990	1.066 (0.685-1.66)	0.790	0.976	1.119 (0.783-1.599)	0.548	0.973
LMF_Chol_IDL	0.893 (0.607-1.313)	0.596	0.955	0.969 (0.75-1.252)	0.811	0.990	1.049 (0.701-1.569)	0.827	0.976	1.337 (0.96-1.863)	0.092	0.973
LMF_Chol_LDL	0.871 (0.586-1.295)	0.528	0.955	1.032 (0.777-1.372)	0.827	0.990	0.964 (0.648-1.433)	0.865	0.976	0.906 (0.63-1.305)	0.610	0.973
LMF_Chol_HDL	1.121 (0.738-1.703)	0.621	0.955	0.875 (0.665-1.152)	0.342	0.990	1.242 (0.776-1.99)	0.411	0.976	1.209 (0.841-1.739)	0.320	0.973
LMF_FreeChol_VLDL	0.602 (0.399-0.91)	0.023	0.301	0.976 (0.735-1.297)	0.868	0.990	1.221 (0.781-1.909)	0.404	0.976	1.141 (0.791-1.646)	0.490	0.973
LMF_FreeChol_IDL	0.873 (0.598-1.274)	0.520	0.955	0.938 (0.708-1.244)	0.659	0.990	0.976 (0.659-1.446)	0.910	0.985	1.277 (0.921-1.772)	0.150	0.973
LMF_FreeChol_LDL	1.29 (0.843-1.971)	0.283	0.872	1.01 (0.755-1.35)	0.947	0.990	0.991 (0.649-1.515)	0.970	0.992	1.106 (0.764-1.601)	0.607	0.973
LMF_FreeChol_HDL	1.352 (0.863-2.12)	0.226	0.822	0.905 (0.688-1.191)	0.479	0.990	1.606 (0.977-2.639)	0.088	0.976	1.308 (0.893-1.914)	0.179	0.973
LMF_Phosp_VLDL	0.525 (0.339-0.814)	0.006	0.301	0.858 (0.639-1.151)	0.307	0.990	1.159 (0.735-1.827)	0.548	0.976	1.126 (0.778-1.628)	0.540	0.973
LMF_Phosp_IDL	0.799 (0.528-1.21)	0.331	0.920	0.918 (0.69-1.223)	0.561	0.990	0.898 (0.582-1.386)	0.660	0.976	1.05 (0.75-1.47)	0.783	0.979
LMF_Phosp_LDL	1.068 (0.714-1.598)	0.767	0.955	1.11 (0.825-1.493)	0.491	0.990	0.907 (0.597-1.377)	0.668	0.976	0.965 (0.668-1.394)	0.855	0.979
LMF_Phosp_HDL	1.023 (0.662-1.581)	0.926	0.987	1.019 (0.779-1.333)	0.894	0.990	0.932 (0.581-1.494)	0.790	0.976	1.216 (0.849-1.742)	0.302	0.973
LMF_ApoA1_HDL	0.851 (0.575-1.261)	0.452	0.955	0.918 (0.691-1.218)	0.553	0.990	1.037 (0.691-1.558)	0.871	0.976	1.171 (0.834-1.645)	0.370	0.973

LMF_ApoA2_HDL	1.058 (0.703-1.592)	0.807	0.955	0.947 (0.713-1.258)	0.709	0.990	0.938 (0.617-1.427)	0.783	0.976	1.249 (0.883-1.766)	0.222	0.973
LMF_ApoB_VLDL	0.597 (0.39-0.914)	0.024	0.301	0.905 (0.688-1.191)	0.478	0.990	1.095 (0.687-1.747)	0.723	0.976	1.044 (0.724-1.505)	0.822	0.979
LMF_ApoB_IDL	0.87 (0.591-1.282)	0.516	0.955	1.03 (0.784-1.354)	0.831	0.990	0.917 (0.603-1.395)	0.708	0.976	1.269 (0.904-1.78)	0.177	0.973
LMF_ApoB_LDL	1.054 (0.709-1.566)	0.811	0.955	0.944 (0.704-1.265)	0.698	0.990	0.918 (0.601-1.402)	0.714	0.976	1.025 (0.709-1.483)	0.898	0.985
SubTrigl_VLDL-1	0.727 (0.468-1.129)	0.186	0.772	0.842 (0.642-1.106)	0.216	0.990	1.113 (0.716-1.729)	0.657	0.976	1.045 (0.733-1.49)	0.815	0.979
SubTrigl_VLDL-2	0.608 (0.392-0.945)	0.041	0.339	0.839 (0.632-1.114)	0.224	0.990	1.064 (0.659-1.718)	0.816	0.976	0.986 (0.68-1.431)	0.944	0.985
SubTrigl_VLDL-3	0.643 (0.406-1.02)	0.082	0.435	1.012 (0.762-1.344)	0.935	0.990	1.114 (0.677-1.834)	0.705	0.976	1.042 (0.719-1.51)	0.836	0.979
SubTrigl_VLDL-4	0.602 (0.372-0.973)	0.047	0.339	1.236 (0.928-1.648)	0.146	0.990	1.159 (0.735-1.828)	0.571	0.976	1.06 (0.755-1.488)	0.741	0.979
SubTrigl_VLDL-5	0.734 (0.478-1.126)	0.190	0.772	1.194 (0.902-1.579)	0.214	0.990	1.359 (0.922-2.002)	0.165	0.976	0.952 (0.691-1.312)	0.768	0.979
SubChol_VLDL-1	0.704 (0.464-1.068)	0.121	0.573	0.921 (0.691-1.228)	0.575	0.990	1.101 (0.712-1.704)	0.684	0.976	1.186 (0.824-1.706)	0.368	0.973
SubChol_VLDL-2	0.554 (0.366-0.838)	0.009	0.301	0.914 (0.692-1.207)	0.524	0.990	1.018 (0.626-1.654)	0.949	0.985	1.075 (0.746-1.55)	0.708	0.979
SubChol_VLDL-3	0.631 (0.406-0.981)	0.055	0.339	0.989 (0.745-1.314)	0.942	0.990	1.119 (0.707-1.771)	0.657	0.976	1.224 (0.857-1.747)	0.278	0.973
SubChol_VLDL-4	0.667 (0.453-0.982)	0.056	0.339	1.119 (0.849-1.475)	0.425	0.990	0.966 (0.647-1.441)	0.873	0.976	1.085 (0.779-1.511)	0.636	0.973
SubChol_VLDL-5	0.841 (0.556-1.271)	0.448	0.955	1.051 (0.793-1.392)	0.733	0.990	0.775 (0.483-1.243)	0.327	0.976	0.885 (0.618-1.266)	0.518	0.973
SubFreeChol_VLDL-1	0.57 (0.363-0.896)	0.020	0.301	1.006 (0.76-1.331)	0.965	0.990	1.025 (0.647-1.623)	0.924	0.985	1.02 (0.72-1.445)	0.912	0.985
SubFreeChol_VLDL-2	0.676 (0.443-1.031)	0.090	0.446	0.972 (0.735-1.285)	0.842	0.990	1.08 (0.683-1.71)	0.764	0.976	1.1 (0.773-1.564)	0.607	0.973
SubFreeChol_VLDL-3	0.661 (0.425-1.029)	0.084	0.435	1.006 (0.754-1.343)	0.969	0.990	1.156 (0.715-1.87)	0.582	0.976	1.277 (0.882-1.85)	0.208	0.973
SubFreeChol_VLDL-4	0.777 (0.538-1.124)	0.218	0.822	1.069 (0.814-1.404)	0.631	0.990	1.054 (0.711-1.563)	0.804	0.976	1.236 (0.89-1.717)	0.212	0.973
SubFreeChol_VLDL-5	0.719 (0.471-1.097)	0.157	0.714	0.986 (0.742-1.311)	0.925	0.990	1.323 (0.861-2.034)	0.242	0.976	0.897 (0.641-1.254)	0.533	0.973
SubPhosp_VLDL-1	0.63 (0.401-0.989)	0.061	0.345	0.898 (0.677-1.19)	0.455	0.990	1.131 (0.704-1.816)	0.637	0.976	1.13 (0.781-1.636)	0.530	0.973
SubPhosp_VLDL-2	0.501 (0.323-0.777)	0.004	0.301	1.008 (0.765-1.328)	0.957	0.990	1.125 (0.664-1.906)	0.691	0.976	1.068 (0.731-1.561)	0.740	0.979
SubPhosp_VLDL-3	0.534 (0.332-0.86)	0.014	0.301	1.027 (0.768-1.372)	0.858	0.990	1.059 (0.645-1.738)	0.838	0.976	1.149 (0.798-1.653)	0.471	0.973
SubPhosp_VLDL-4	0.627 (0.412-0.952)	0.039	0.339	1.179 (0.878-1.584)	0.272	0.990	0.961 (0.623-1.484)	0.869	0.976	1.043 (0.737-1.475)	0.817	0.979
SubPhosp_VLDL-5	0.74 (0.502-1.091)	0.165	0.723	1.058 (0.797-1.405)	0.698	0.990	0.957 (0.625-1.467)	0.853	0.976	0.862 (0.614-1.209)	0.403	0.973
SubTrigl_LDL-1	0.939 (0.633-1.392)	0.776	0.955	0.962 (0.721-1.284)	0.794	0.990	0.576 (0.357-0.927)	0.041	0.934	1.017 (0.716-1.444)	0.929	0.985
SubTrigl_LDL-2	1.007 (0.668-1.518)	0.977	0.997	1.069 (0.81-1.41)	0.638	0.990	0.567 (0.353-0.912)	0.039	0.934	1.048 (0.73-1.504)	0.813	0.979
SubTrigl_LDL-3	1 (0.65-1.538)	0.999	0.999	0.832 (0.631-1.097)	0.187	0.990	0.859 (0.533-1.383)	0.567	0.976	1.105 (0.774-1.578)	0.609	0.973
SubTrigl_LDL-4	0.799 (0.502-1.272)	0.393	0.955	0.866 (0.661-1.135)	0.294	0.990	0.755 (0.439-1.297)	0.370	0.976	0.998 (0.694-1.436)	0.991	0.991
SubTrigl_LDL-5	0.783 (0.531-1.154)	0.261	0.826	0.787 (0.589-1.054)	0.102	0.990	0.717 (0.455-1.131)	0.207	0.976	1.095 (0.788-1.522)	0.603	0.973
SubTrigl_LDL-6	0.999 (0.651-1.531)	0.995	0.999	0.962 (0.732-1.263)	0.779	0.990	1.478 (0.93-2.35)	0.151	0.976	1.075 (0.752-1.537)	0.704	0.979
SubChol_LDL-1	1.107 (0.731-1.676)	0.662	0.955	0.934 (0.717-1.215)	0.609	0.990	0.826 (0.504-1.351)	0.490	0.976	1.07 (0.752-1.521)	0.719	0.979

SubChol_LDL-2	1.083 (0.729-1.61)	0.716	0.955	0.85 (0.637-1.136)	0.271	0.990	0.953 (0.616-1.473)	0.841	0.976	0.951 (0.682-1.325)	0.771	0.979
SubChol_LDL-3	1.081 (0.695-1.68)	0.754	0.955	0.971 (0.743-1.27)	0.833	0.990	0.84 (0.525-1.343)	0.501	0.976	0.926 (0.633-1.354)	0.703	0.979
SubChol_LDL-4	0.803 (0.537-1.201)	0.321	0.914	0.933 (0.698-1.248)	0.642	0.990	0.97 (0.633-1.484)	0.896	0.985	0.934 (0.664-1.313)	0.702	0.979
SubChol_LDL-5	0.849 (0.579-1.246)	0.437	0.955	0.97 (0.73-1.289)	0.834	0.990	0.819 (0.56-1.197)	0.335	0.976	0.791 (0.569-1.099)	0.165	0.973
SubChol_LDL-6	0.989 (0.677-1.445)	0.958	0.997	1.156 (0.882-1.514)	0.295	0.990	1.209 (0.786-1.86)	0.417	0.976	1.136 (0.809-1.594)	0.484	0.973
SubFreeChol_LDL-1	1.159 (0.775-1.735)	0.512	0.955	1.006 (0.764-1.323)	0.969	0.990	0.816 (0.508-1.311)	0.458	0.976	1.1 (0.782-1.548)	0.595	0.973
SubFreeChol_LDL-2	1.099 (0.763-1.583)	0.635	0.955	0.913 (0.688-1.212)	0.530	0.990	1.08 (0.701-1.665)	0.751	0.976	0.993 (0.717-1.376)	0.967	0.985
SubFreeChol_LDL-3	1.385 (0.87-2.205)	0.231	0.822	0.906 (0.693-1.183)	0.467	0.990	0.928 (0.585-1.471)	0.776	0.976	1.009 (0.699-1.454)	0.965	0.985
SubFreeChol_LDL-4	0.94 (0.606-1.457)	0.801	0.955	0.907 (0.677-1.216)	0.515	0.990	0.945 (0.614-1.455)	0.812	0.976	0.894 (0.625-1.279)	0.553	0.973
SubFreeChol_LDL-5	0.919 (0.621-1.36)	0.701	0.955	0.913 (0.688-1.211)	0.529	0.990	0.851 (0.588-1.231)	0.424	0.976	0.901 (0.657-1.237)	0.528	0.973
SubFreeChol_LDL-6	1.067 (0.703-1.619)	0.778	0.955	0.977 (0.735-1.299)	0.875	0.990	0.901 (0.598-1.356)	0.640	0.976	0.918 (0.65-1.298)	0.640	0.973
SubPhosp_LDL-1	1.095 (0.723-1.66)	0.697	0.955	0.944 (0.722-1.235)	0.677	0.990	0.795 (0.483-1.307)	0.413	0.976	1.077 (0.759-1.529)	0.691	0.979
SubPhosp_LDL-2	1.074 (0.713-1.618)	0.756	0.955	0.979 (0.743-1.29)	0.882	0.990	0.743 (0.457-1.207)	0.276	0.976	1.007 (0.714-1.421)	0.968	0.985
SubPhosp_LDL-3	1.266 (0.788-2.033)	0.381	0.955	0.945 (0.723-1.235)	0.677	0.990	0.793 (0.502-1.253)	0.354	0.976	1.015 (0.696-1.481)	0.941	0.985
SubPhosp_LDL-4	0.872 (0.568-1.339)	0.566	0.955	0.852 (0.627-1.158)	0.302	0.990	1.025 (0.64-1.643)	0.924	0.985	1.071 (0.737-1.555)	0.729	0.979
SubPhosp_LDL-5	0.872 (0.586-1.297)	0.535	0.955	0.836 (0.626-1.116)	0.224	0.990	0.804 (0.542-1.193)	0.314	0.976	0.84 (0.602-1.172)	0.311	0.973
SubPhosp_LDL-6	0.978 (0.656-1.456)	0.917	0.987	1.133 (0.855-1.5)	0.386	0.990	1.286 (0.819-2.02)	0.311	0.976	1.144 (0.806-1.622)	0.469	0.973
SubApoB_LDL-1	1.092 (0.728-1.638)	0.698	0.955	0.964 (0.726-1.28)	0.801	0.990	0.864 (0.525-1.421)	0.612	0.976	1.161 (0.819-1.646)	0.420	0.973
SubApoB_LDL-2	1.023 (0.687-1.525)	0.917	0.987	0.917 (0.688-1.223)	0.558	0.990	0.764 (0.468-1.249)	0.335	0.976	0.956 (0.681-1.344)	0.802	0.979
SubApoB_LDL-3	1.236 (0.8-1.912)	0.389	0.955	0.875 (0.668-1.146)	0.330	0.990	0.726 (0.458-1.149)	0.193	0.976	1.034 (0.711-1.502)	0.867	0.979
SubApoB_LDL-4	0.769 (0.505-1.171)	0.256	0.826	0.873 (0.646-1.18)	0.376	0.990	1.192 (0.743-1.91)	0.506	0.976	1.093 (0.764-1.563)	0.637	0.973
SubApoB_LDL-5	0.837 (0.57-1.228)	0.400	0.955	0.765 (0.567-1.032)	0.076	0.990	0.762 (0.519-1.12)	0.190	0.976	0.846 (0.606-1.182)	0.335	0.973
SubApoB_LDL-6	0.971 (0.659-1.428)	0.887	0.982	1.01 (0.766-1.331)	0.944	0.990	1.311 (0.83-2.069)	0.274	0.976	1.197 (0.846-1.694)	0.332	0.973
SubTrigl_HDL-1	0.88 (0.594-1.302)	0.562	0.955	0.996 (0.752-1.318)	0.975	0.990	0.887 (0.54-1.457)	0.683	0.976	1.193 (0.857-1.661)	0.307	0.973
SubTrigl_HDL-2	0.773 (0.535-1.116)	0.204	0.802	0.87 (0.652-1.162)	0.347	0.990	0.821 (0.533-1.264)	0.426	0.976	1.023 (0.747-1.401)	0.889	0.985
SubTrigl_HDL-3	0.626 (0.43-0.912)	0.019	0.301	0.877 (0.668-1.15)	0.340	0.990	0.737 (0.481-1.13)	0.220	0.976	1.003 (0.736-1.367)	0.986	0.991
SubTrigl_HDL-4	0.656 (0.449-0.959)	0.037	0.339	0.874 (0.659-1.158)	0.344	0.990	0.634 (0.429-0.937)	0.040	0.934	0.823 (0.59-1.147)	0.267	0.973
SubChol_HDL-1	0.992 (0.661-1.487)	0.971	0.997	0.972 (0.742-1.275)	0.839	0.990	1.497 (0.909-2.465)	0.176	0.976	1.315 (0.936-1.848)	0.130	0.973
SubChol_HDL-2	1.094 (0.767-1.56)	0.658	0.955	1.386 (1.026-1.872)	0.028	0.990	1.207 (0.75-1.943)	0.523	0.976	1.276 (0.929-1.754)	0.141	0.973
SubChol_HDL-3	1.149 (0.743-1.776)	0.570	0.955	0.915 (0.699-1.198)	0.518	0.990	0.983 (0.613-1.577)	0.950	0.985	1.265 (0.879-1.821)	0.215	0.973
SubChol_HDL-4	1.095 (0.748-1.602)	0.672	0.955	0.771 (0.57-1.042)	0.074	0.990	1.112 (0.754-1.642)	0.613	0.976	1.165 (0.836-1.624)	0.409	0.973

SubFreeChol_HDL-1	1.148 (0.735-1.793)	0.579	0.955	1.009 (0.761-1.338)	0.951	0.990	1.234 (0.767-1.985)	0.420	0.976	1.252 (0.858-1.828)	0.257	0.973
SubFreeChol_HDL-2	1.273 (0.84-1.928)	0.295	0.884	1.182 (0.902-1.548)	0.219	0.990	0.997 (0.639-1.556)	0.991	0.992	1.405 (0.973-2.027)	0.073	0.973
SubFreeChol_HDL-3	1.144 (0.72-1.819)	0.625	0.955	0.885 (0.664-1.18)	0.406	0.990	0.87 (0.569-1.331)	0.578	0.976	1.16 (0.82-1.642)	0.411	0.973
SubFreeChol_HDL-4	1.077 (0.707-1.64)	0.757	0.955	0.815 (0.615-1.081)	0.156	0.990	1.107 (0.758-1.616)	0.628	0.976	1.097 (0.797-1.508)	0.581	0.973
SubPhosp_HDL-1	0.994 (0.659-1.5)	0.980	0.997	1.058 (0.806-1.388)	0.686	0.990	1.396 (0.832-2.342)	0.290	0.976	1.367 (0.979-1.91)	0.076	0.973
SubPhosp_HDL-2	1.084 (0.736-1.596)	0.719	0.955	1.117 (0.852-1.463)	0.423	0.990	1.024 (0.617-1.702)	0.936	0.985	1.292 (0.943-1.769)	0.121	0.973
SubPhosp_HDL-3	0.964 (0.618-1.502)	0.882	0.982	0.861 (0.654-1.135)	0.283	0.990	0.738 (0.441-1.236)	0.297	0.976	1.224 (0.845-1.772)	0.300	0.973
SubPhosp_HDL-4	0.927 (0.6-1.432)	0.758	0.955	0.799 (0.6-1.063)	0.115	0.990	0.927 (0.576-1.493)	0.776	0.976	1.042 (0.719-1.509)	0.839	0.979
SubApoA1_HDL-1	0.96 (0.682-1.352)	0.828	0.963	1.024 (0.771-1.36)	0.872	0.990	1.072 (0.714-1.611)	0.762	0.976	1.113 (0.82-1.509)	0.504	0.973
SubApoA1_HDL-2	0.935 (0.606-1.442)	0.782	0.955	1.26 (0.956-1.661)	0.095	0.990	0.997 (0.646-1.538)	0.989	0.992	1.207 (0.852-1.711)	0.306	0.973
SubApoA1_HDL-3	0.94 (0.612-1.444)	0.796	0.955	0.937 (0.724-1.212)	0.620	0.990	1.095 (0.696-1.722)	0.717	0.976	1.269 (0.888-1.812)	0.200	0.973
SubApoA1_HDL-4	0.904 (0.604-1.353)	0.649	0.955	1.002 (0.757-1.325)	0.990	0.990	1.089 (0.734-1.617)	0.689	0.976	1.018 (0.712-1.455)	0.925	0.985
SubApoA2_HDL-1	0.942 (0.603-1.47)	0.813	0.955	1.012 (0.762-1.342)	0.937	0.990	1.151 (0.713-1.859)	0.606	0.976	1.38 (0.978-1.946)	0.044	0.973
SubApoA2_HDL-2	0.96 (0.624-1.475)	0.865	0.982	1.237 (0.935-1.636)	0.135	0.990	1.056 (0.666-1.674)	0.832	0.976	1.419 (1.004-2.006)	0.042	0.973
SubApoA2_HDL-3	0.918 (0.605-1.394)	0.716	0.955	0.892 (0.675-1.177)	0.419	0.990	0.774 (0.491-1.22)	0.311	0.976	1.277 (0.899-1.815)	0.184	0.973
SubApoA2_HDL-4	1.112 (0.747-1.656)	0.631	0.955	0.914 (0.692-1.208)	0.530	0.990	1.026 (0.703-1.497)	0.900	0.985	1.108 (0.796-1.542)	0.560	0.973
